# Emergence and phylogeography of the dengue vector *Aedes aegypti* in Southeastern Iran

**DOI:** 10.1371/journal.pntd.0014671

**Published:** 2026-08-20

**Authors:** Jalil Nejati, Mona Koosha, Nayyereh Choubdar, Sara Rahimy, Hassan Balouch, Mohammad Ali Oshaghi

**Affiliations:** 1 Health Promotion Research Center, Zahedan University of Medical Sciences, Zahedan, Iran; 2 Department of Medical Parasitology and Mycology, School of Medicine, Shahid Beheshti University of Medical Sciences, Tehran, Iran; 3 Department of Vector Biology and Control of Diseases, School of Public Health, Tehran University of Medical Sciences, Tehran, Iran; 4 Center for Disease Control and Prevention, Health Deputy, Iranshahr University of Medical Sciences, Iranshahr, Iran; Universita degli Studi di Pavia, ITALY

## Abstract

**Background:**

*Aedes (Stegomyia) aegypti* (Linnaeus) is the primary vector of dengue, chikungunya, Zika, and yellow fever viruses. Its recent detection in southeastern Iran raises public health concerns about arbovirus spread to new regions. This study provides the first genetic and phylogeographic analysis of *Ae. aegypti* populations from Sistan and Baluchistan Province (SBP), Iran, to infer their origin and invasion pathways.

**Methods:**

Mitochondrial COI and ND4 genes were analysed in newly collected *Ae. aegypti* specimens from border areas, ports, and urban centres of SBP. Haplotype network analyses were constructed using the TCS method in PopART, and phylogenetic analyses were conducted using global reference sequences.

**Results:**

Iranian specimens comprised 7 COI haplotypes (n = 18) and 10 ND4 haplotypes (n = 17). COI phylogeny placed Iranian specimens into two main clades, while ND4 analysis distributed them across several derived clades, mostly clustering with lineages from Latin America (Brazil, Mexico) or Africa. One Iranian specimen showed a close relationship with a Saudi Arabian sequence (bootstrap: 98%) near the basal region. Combined COI + ND4 analysis revealed a monophyletic clade of Iranian specimens with a Sri Lankan specimen, distinct from other global lineages. The global COI network (n = 47) showed a star-like topology with a dominant haplotype 1 shared among 10 Iranian specimens. The ND4 network (n = 31) revealed a complex topology with 18 haplotypes, where a Saudi Arabian and one Iranian specimen (~30 mutational steps) possibly represented the peripheral root.

**Conclusions:**

Detection of diverse *Ae. aegypti* clades confirm establishment of this vector in southeastern Iran. Results support multiple introductions and genetic connectivity with Latin America, Africa, and South Asia, pointing to an emerging invasion corridor. Continued genomic surveillance and integrated vector monitoring are urgently needed to guide prevention strategies.

## Author summary

*Aedes aegypti* is the primary vector of dengue, chikungunya, Zika, and yellow fever viruses. Although its distribution has long been restricted to tropical and subtropical regions, its recent detection in southeastern Iran raises new concerns about the potential spread of arboviruses to previously unaffected areas. In this study, we analyzed mitochondrial COI and ND4 sequences and constructed haplotype networks to investigate the genetic relationships of *Ae. aegypti* collected from border regions, ports, and urban centers in Sistan and Baluchistan Province, Iran. We identified two distinct mitochondrial lineages through COI phylogeny and multiple derived clades through ND4 analysis, with Iranian specimens clustering with lineages from Latin America (Brazil, Mexico) and Africa. One Iranian specimen showed a close genetic relationship with a Saudi Arabian sequence near the root of the ND4 network, while combined COI + ND4 analysis placed Iranian specimens with a Sri Lankan lineage, forming a monophyletic clade distinct from other global populations. These findings suggest that, *Ae. aegypti* has been introduced into Iran multiple times, most likely via Chabahar Port and maritime trade routes, with genetic connections spanning three continents. Our results confirm the establishment of *Ae. aegypti* populations in southeastern Iran and highlight the risk that dengue and other arboviruses could gain a new foothold in this region, emphasizing the need for continued surveillance and vector control strategies.

## Introduction

Over the past few decades, several mosquito-borne viruses, including dengue, Zika, and chikungunya, have emerged or re-emerged in many regions worldwide [1]. Dengue fever (DF) is an arboviral disease affecting humans in tropical areas, with over 2.5 billion people at risk of infection. It is estimated that 50–100 million new infections occur annually, with an average fatality rate for dengue hemorrhagic fever (DHF) ranging from 5% to 40% [2].

Dengue transmission occurs in two separate ecological cycles: an enzootic cycle in forested environments and an urban cycle, which involves humans and mosquitoes, such as *Aedes aegypti* and *Ae. albopictus* [3]. *Aedes aegypti*, the primary epidemic vector for dengue, is believed to originate from Africa [4]. This species likely invaded Asia in the latter half of the 19th century, facilitated by expanding maritime trade and shipping routes [3]. It is a polytypic species comprising two main forms: Ae. aegypti aegypti (Aaa), a light-colored form that breeds in domestic or peridomestic environments and is prevalent in the New World, Asia, coastal East Africa, and *Ae. aegypti* formosus (Aaf), a darker form found primarily in sub-Saharan Africa, breeding in tree and rock holes. Dengue epidemics in the Americas and Asia are associated exclusively with *Ae. aegypti* [2]. This species is highly adapted to urban areas and is typically found near human populations [4]. Urban growth in tropical developing countries, combined with its anthropophilic behavior, the ability to lay desiccation-resistant eggs in artificial containers, and global transportation, has contributed to the geographical spread of this vector, facilitating the spread of dengue [5].

Recent shifts in dengue transmission patterns—from urban epidemics to endemic transmission—have been linked to the genetic characteristics of *Ae. aegypti* [3]. Additionally, in the absence of an effective vaccine, dengue control relies heavily on vector management, particularly the use of insecticides, which significantly influences the genetic variability of this species [5]. Genetic variability and vector competence for the dengue virus have also been observed among closely located *Ae*. *aegypti* populations, highlighting the importance of conducting genetic studies on this species [6].

Studies on the global colonization history, vector competence, and genetic structure of *Ae*. *aegypti* using various nuclear and mitochondrial markers have shown that the species comprises two genetic groups or clades corresponding to its two recognized subspecies [7]. The first group consists of *Aedes aegypti aegypti* (Aaa) populations from East Africa, South America, and the Caribbean. This subspecies has a lighter body color with pale abdominal scales, breeds preferentially in artificial containers associated with human dwellings, and is highly anthropophilic. It is thought to have derived from East African ancestors and has since spread globally, becoming the primary vector of dengue, yellow fever, Zika, and chikungunya viruses [7,8]. The second group contains *Aedes aegypti formosus* (Aaf) populations from Asia and the southeastern United States, with a basal branch containing *Aaf* from East and West Africa. In contrast to *Aaa*, this subspecies has a darker body color lacking pale scales on the first abdominal segment, typically breeds in natural containers such as tree holes in forested environments, and exhibits generalist feeding behavior with lower vector competence for human arboviruses [7,9]. While intermediate or hybrid populations exhibiting mixed *Aaa/Aaf* traits have been documented in East Africa [10], such populations are not known to occur in Asia. Human movement and trade are believed to have facilitated these recent divergences, enabling *Ae. aegypti* to spread from Africa to the Americas and later to Asia [5,11]. Therefore, studying the genetic separation of these two clades is epidemiologically important, as populations from different origins vary markedly in vector competency for arboviruses and insecticide resistance [7,12,13]. Experimentally, *Aaa* strains have shown infection rates of 80% and transmission rates of 43% for yellow fever virus, whereas Aaf from the same region exhibited only 26% infection and 7% transmission [14]. Similarly, studies on dengue virus in West Africa have found disseminated infection rates ranging from 0-100% depending on the specific mosquito population and viral strain, with *Aaf* populations often, but not always, showing lower competence than *Aaa* [15]. These differences, which can exceed 10-fold for some virus-vector combinations, highlight the epidemiological significance of distinguishing between the two subspecies and their local populations.

Mitochondrial genes, such as cytochrome oxidase subunit I (*COI*) and nicotinamide adenine dinucleotide (NADH) dehydrogenase subunit 4 (*ND4*), were selected as molecular markers due to their high mutation rates and phylogeographic utility [16–18]. These genes are powerful tools for reconstructing evolutionary relationships within species, given their maternal inheritance, high copy number, sequence variability, and lack of recombination [19–22]. Their rapid mutation rates and ability to resolve ancestral lineages make them particularly valuable for inferring population structures, divergence, and migration patterns [6,16–18].

Historical records of dengue in Iran date back to 2008, when the first imported case was documented [23]. Following this event, specimens of *Aedes albopictus* were collected from several southeastern counties, including Lashar and Rask in 2009 and Chabahar County (Vashnam and Paroomi villages) in 2013, which raised public health concerns [24]. However, subsequent investigations did not verify the establishment of *Ae. albopictus* in either the southeastern counties or the northern counties [25]. Separately, *Ae. albopictus* has also been detected in northern Iran in 2025 [26], suggesting establishment in northern and western parts of the country. Regarding the other dengue vector, *Aedes aegypti*, historical evidence of its presence in southern Iran extends back more than 50 years, with records from 1920, 1921, 1951, and 1953 in the southwestern and southern regions of the country [27]. Nevertheless, as with *Ae. albopictus*, no confirmed detections had been reported since then. Considering its presence in Saudi Arabia, the potential re‑emergence of *Ae. aegypti* in southern Iran had been anticipated [23], and this prediction was substantiated by the detection of the species in Hormozgan Province in 2020 [28]. Its possible expansion into southeastern areas bordering Pakistan, a country with recurrent dengue outbreaks, is a significant concern. Studying the genetic diversity and phylogeny of local *Ae. aegypti* populations is crucial because genetic variation can affect vector competence for arboviruses and insecticide resistance, both of which directly influence disease transmission and control strategies [7,12,13].

This study aimed to determine the distribution and genetic diversity of *Ae. aegypti* across a wide geographic area in southeastern Iran and to confirm species identification using molecular analysis. Furthermore, we assessed the phylogenetic relatedness of Iranian *Ae. aegypti* populations with global reference sequences. By combining field surveillance with genetic characterization, we sought to provide a comprehensive understanding of the establishment, origin, and population structure of this vector in an emerging invasion corridor.

## Methods

### Ethical statement

This study was approved by the Ethics Health Promotion Research Center, Zahedan University of Medical Sciences (IR.ZAUMS.REC.1402.333). Verbal informed consent was obtained from homeowners or property owners prior to placing ovitraps inside homes or other private locations. All data were anonymized to protect participant privacy.

### Study sites

**Fig 1:**
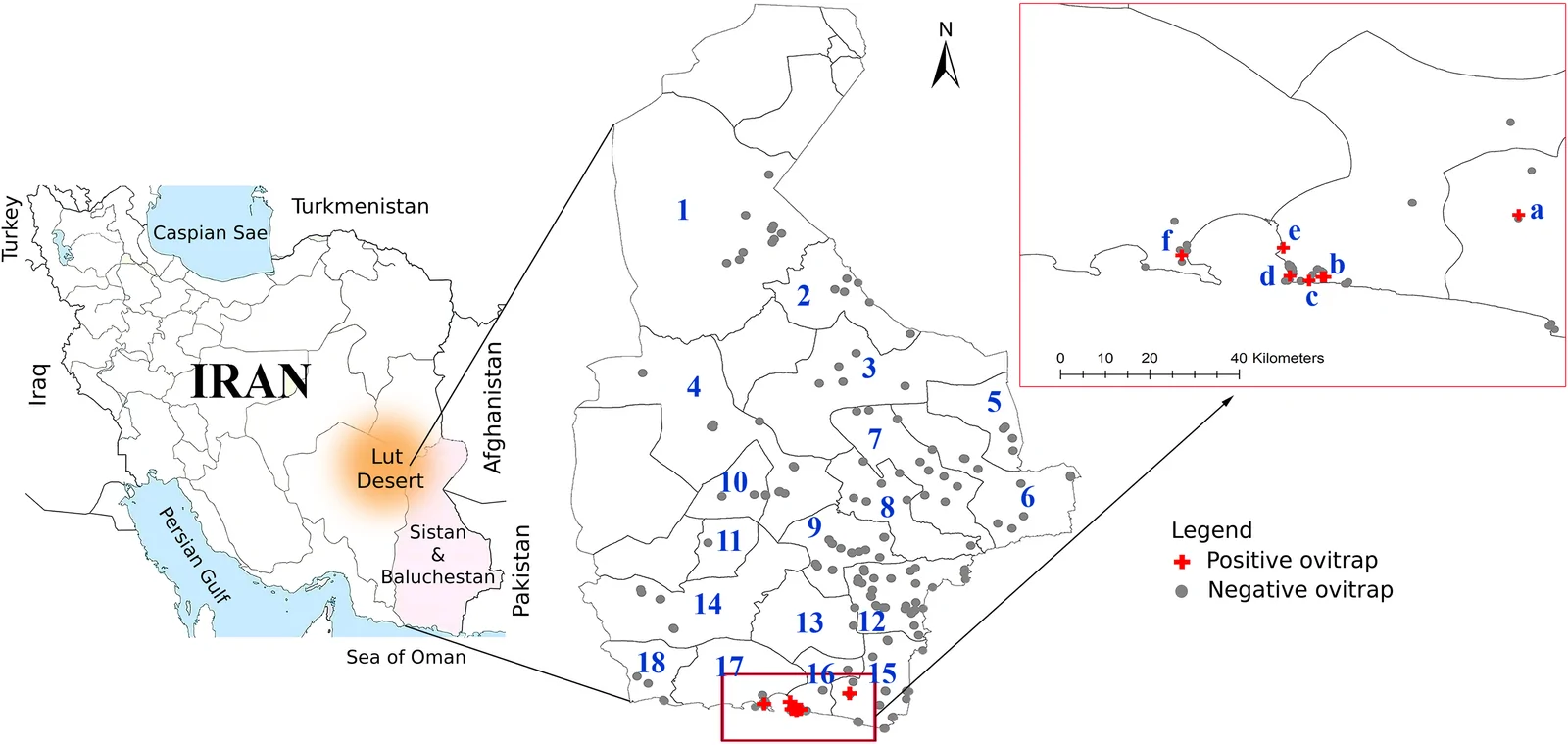
Location of the study area and ovitrap sampling sites in Sistan and Baluchistan Province, southeastern Iran, 2023–2024. Numbers and letters indicate sampling locations: 1. Zahedan; 2. Mirjaveh; 3. Khash; 4. Iranshahr; 5. Golshan; 6. Saravan; 7. Sib-va-suran; 8. Mehrestan; 9. Sarbaz; 10. Bampour; 11. Lashar; 12. Rask; 13. Ghasr-e-ghand; 14. Nikshahr; 15. Dashtyari; 16. Chabahar; 17. Konarak; 18. Zarabad; a. Negur; b. Komb Suburb, Golshahr and Zibashahr residential areas; c. Beltway and Ghods Blvd.; d. Bazar and Tohid Blvd.; e. Tis and Small Sea; f. Konarak. Positive and negative ovitraps for *Ae. aegypti* are shown by red and blue dots, respectively. The location of the Lut Desert is shown as a brown spot color on the map. Country outline map adapted from Choubdar et al. (2021), https://doi.org/10.1371/journal.pntd.0009480, published under CC BY 4.0 (https://creativecommons.org/licenses/by/4.0/). Province boundary shapefile from Natural Earth (https://www.naturalearthdata.com/), public domain.

This study was conducted from April 2023 to June 2024 across eighteen counties in Sistan and Baluchistan Province, located in southeastern Iran (Fig 1). Table 1 provides detailed collection site information for the eleven counties where *Ae. aegypti* was detected. The province shares borders with Pakistan and Afghanistan, where malaria and dengue are prevalent. Geographically, it spans ~25.09°–31.44° N latitude and ~58.78°–63.26° E longitude, encompassing diverse climates.

**Table 1:** *Aedes aegypti* samples collected from different locations in Sistan and Baluchistan Province, Iran, 2023-2024.

No.	County	Collection sites	Geographical coordinates*		No. of samples
			Longitude	Latitude	
1	Chabahar	Bazar	E 60.6440	N 25.3062	20
2		Ghods Blvd.	E 60.6679	N 25.2834	14
3		Small Sea	E 60.6273	N 25.2971	12
4		Zibashahr Residential Area	E 60.6972	N 25.2897	14
5		Komb Suburb	E 60.7039	N 25.2897	15
6		City of Tis	E 60.6175	N 25.3609	14
7		Golshahr Residential Area	E 60.6756	N 25.2862	10
8		Tohid Blvd.	E 60.6611	N 25.2964	13
9		Beltway	E 60.6559	N 25.2988	12
10	Konarak	City of Konarak	E 60.3931	N 25.3588	20
11	Dashtyari	City of Negur	E 61.1402	N 25.4000	20
Total					164

*: Note: Coordinates are given to four decimal places (precision ~11 m). Ovitraps at each collection site were placed within a maximum of 10 meters from the recorded coordinates.

The northern and central regions, influenced by the Lut Desert (the second largest desert of Iran, located between the provinces of Khorasan, Kerman, and Yazd, Fig 1), have an arid climate, with average annual rainfall of approximately 100 mm and summer temperatures that can exceed 40°C; the highlands may receive occasional snowfall. In contrast, the southern regions experience a subtropical climate with relative humidity ranging from 50% to 95%, characterized by monsoon winds and summer rainfall that create favorable conditions for vector proliferation and malaria transmission [29,30]. The province’s average temperatures generally range from 22°C to 37°C, with annual precipitation varying from ~178 mm in many areas to ~280 mm in the southwestern region. Recorded extreme temperatures range approximately from −16.5°C to 42.4°C, reflecting the climatic diversity across the province [25,31].

### Ovitrap surveillance

Ovitraps, widely recognized as cost-effective and reliable tools for monitoring *Aedes* populations, were used in this study to assess their spatiotemporal distribution and potential risk areas for dengue transmission [32]. These traps also facilitate community-based larval surveillance, contributing to dengue prevention and control efforts.

Ovitrap surveillance was conducted biweekly (once every 14 days) over a 12-month period, beginning the day after ovitraps were set. A team of eight trained technicians installed 50–100 ovitraps per site, depending on the area size and habitat complexity, across 341 sites in the central and southern regions of the province (Fig 1). Trap placement followed the national vector surveillance guideline [33]. This guideline prioritizes high-risk entry points (border zones, customs checkpoints, seaports, and urban centers) for ovitrap deployment to enable early detection of invasive mosquito species. Each ovitrap was filled with 10% wheat-straw infusion prepared with non-chlorinated water to attract gravid female mosquitoes for oviposition.

### Morphological and mtDNA analysis

When ovitraps yielded positive results, eggs and larvae were transferred to the insectary for processing. Some third- and fourth-instar larvae were preserved in lactophenol solution, and collection data were recorded. Morphological identification was performed using standard taxonomic keys [34] under a stereomicroscope (Olympus SZX16, Japan). The remaining larvae were reared to adults, frozen, and identified to species level. Confirmed *Ae. aegypti* specimens were selected for molecular analysis.

For DNA extraction, dehydrated specimens were ground in 1.5 mL Eppendorf tubes using autoclaved glass pestles. DNA was extracted with the G-spin kit (iNtRON Biotechnology, Korea) following the manufacturer’s protocol. DNA concentration and purity were assessed using a NanoDrop spectrophotometer (Thermo Fisher Scientific, USA). Extracted DNA was eluted in 50 μL of elution buffer and stored at −20°C until further use.

Two mitochondrial genes, NADH dehydrogenase subunit 4 (ND4) and cytochrome oxidase subunit I (COI), were targeted to analyze genetic structure and sequence polymorphism. The COI gene (710 bp) was amplified using primers LCO1490 and HCO2198 [35] under the following thermal cycling conditions: initial denaturation at 95°C for 5 min; 5 cycles of 94°C for 40 s, 45°C for 60 s, and 72°C for 60 s; followed by 35 cycles of 94°C for 40 s, 54°C for 60 s, and 72°C for 60 s; and a final extension at 72°C for 10 min. The ND4 gene (344 bp) was amplified using primers F-ND4 and R-ND4 [36] with the following conditions: initial denaturation at 96°C for 5 min; 35 cycles of 94°C for 40 s, 56°C for 40 s, and 72°C for 40 s; and a final extension at 72°C for 5 min.

PCR reactions (25 μL) contained 12.5 μL of PCR Master Mix (Ampliqon, Denmark), 1 μL of each primer (10 μM), 3 μL of template DNA, and 7.5 μL of nuclease-free water. Negative controls were included by omitting genomic DNA. Amplified products were visualized under UV transillumination after electrophoresis on 1.5% agarose gels stained with Nucleic Acid Gel Stain (SMOBiO, Taiwan). Successful amplicons were purified and sequenced by the Sanger method at Genetic Codon Sequencing Service (Tehran, Iran).

### mtDNA data analysis

Sequences were analyzed using BioEdit v7.2.5 and refined in Chromas v2.6.6 by removing low-quality regions. Consensus sequences were compared with GenBank entries using BLAST (https://www.ncbi.nlm.nih.gov/BLAST).

To assess the genealogical relationships among COI haplotypes and visualize phylogeographic patterns, a haplotype network was constructed. The analysis included 47 COI sequences (659 bp): 18 from the present study (Iran) and 29 from global populations retrieved from GenBank (S1 Table). Sequences were aligned using ClustalW [37]. The network was generated using PopART v.1.7 (https://www.popart.otago.ac.nz), which estimates the most parsimonious connections among haplotypes with a 95% parsimony connection limit. Gaps were treated as missing data. The resulting network was visualized with circle sizes proportional to haplotype frequency and branch lengths proportional to the number of mutational steps between haplotypes.

Phylogenetic relationships were assessed to determine whether local haplotypes corresponded to globally reported COI and ND4 haplotypes. Multiple sequence alignments were performed using ClustalW [37]. Phylogenetic trees were constructed using the Maximum Likelihood (ML) method based on Kimura’s two-parameter model in MEGA version 11. Bootstrap analysis (1,000 replicates) was used to evaluate the robustness of the inferred tree topologies.

To provide a global context, we conducted comparative analyses with GenBank sequences selected to maximize geographical representation (populations from the Americas, Europe, Asia, Africa, and Australia) and to capture the broad diversity of the vector’s mitochondrial lineages (S1 and S2 Tables). For phylogenetic reconstruction, *Aedes albopictus* was selected as the outgroup to root the trees [38]. To verify sequence integrity, nucleotide sequences were translated into amino acid sequences using the invertebrate mitochondrial genetic code in ExPASy (https://web.expasy.org).

## Results

### Morphological analysis

During the 14-month study period, *Ae. aegypti* eggs or larvae were detected at 8 of the 253 sampling sites across the study regions. On August 30, 2023, five ovitraps in Chabahar County consistently tested positive during each biweekly monitoring period. The number of ovitraps containing *Aedes* eggs or larvae increased gradually, and by June 2024, two additional sites in Konarak and Negur counties also yielded positive results.

**Fig 2:**
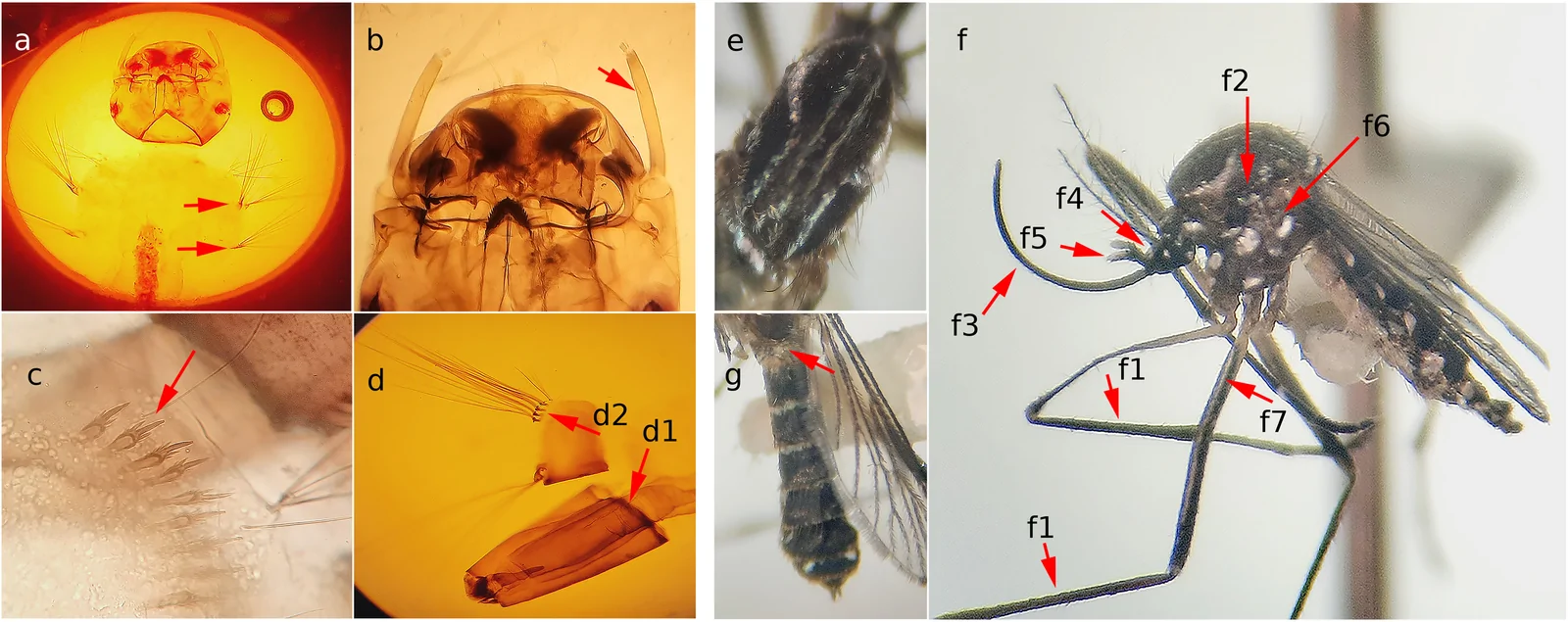
Identification characteristics of *Aedes (Stegomyia) aegypti* larvae and female adults collected from the southeastern corner of Iran, indicated by the red arrow (200X magnification). Left panel (larvae): (a) Basal tubercles of setae 9–12-M, T were highly sclerotized; (b) Antenna was smooth, and seta 1-A was singular; (c) Comb scales featured stout subapical spines; (d) Siphon lacked an acus (d1), and seta 4-X consisted of five pairs of setae, each typically two- or three-branched (d2). Right panel (female adults): (e) Lyre-shaped pattern on the scutum; (f) tibiae lacked median white bands (f1), paratergite had scales (f2), proboscis was approximately the same length as the forefemur (f3), white scale patches were observed on the clypeus (f4), maxillary palpus with white scales (f5), mesepimeron without V-shaped white scale patches (f6), anterior surface of the mid-femur featured a longitudinal white stripe (f7); (g) first abdominal tergite with a large central area of pale scales. All images in this figure are original photographs taken by the first author and are published here with full permission under the CC BY 4.0 license.

Morphological examination confirmed all specimens as *Ae. aegypti* (Linnaeus), representing the first documented record of this species in the southeastern region of Iran. Key diagnostic characteristics are summarized in Fig 2.

Larvae were identified by the absence of an acus on the siphon, the presence of stout subapical spines on the comb scales, and highly sclerotized basal tubercles on setae 9–12-M and T. Adults displayed the characteristic lyre-shaped pattern on the scutum, a trilobed scutellum, and distinctive white scaling patterns, including a longitudinal stripe on the anterior surface of the mid-femur and white scale patches on the clypeus, maxillary palpus, and mesepimeron (Fig 2).

### MtDNA analysis

#### Specimen and sequence summary.

A total of 18 *Ae. aegypti* specimens from 11 localities in Sistan and Baluchistan Province, Iran (Table 1, sites 1–11), were included in the final molecular analysis. We obtained COI sequences from all 18 specimens and ND4 sequences from 17 specimens (one specimen failed to amplify for ND4).

### COI gene analysis

The complete alignment of all fragments spanned 659 nucleotides of the COI gene, comprising seven haplotypes (Table 2). A total of 12 polymorphic sites (1.82%) were identified, all of which were parsimony-informative, with no singleton variable sites detected. These polymorphic sites were distributed almost uniformly along the gene, occurring at positions 57, 192, 258, 261, 270, 327, 483, 492, 504, 525, 603, and 654 (Table 3). Haplotype 1, the most frequent (33.3%; Table 2), was dominant across multiple localities, being identified in six specimens from the study region.

**Table 2:** Haplotype frequency and distribution of *Aedes aegypti* in Sistan and Baluchistan, Iran, based on COI, ND4, and combined mitochondrial genes.

	Length (bp) number of specimens (n)	Haplotype	Number of sequences in each haplotype	Frequency in each haplotype	GenBank ID (Accession numbers) (This study)
**Mitochondrial markers**	**COI**				
	**659** **(n = 18)**	Hap_1	6	0.33333	PQ473699, PQ473700, PQ473702, PQ473704, PQ473705, PQ473709
		Hap_2	3	0.16666	PQ473697, PQ473703, PQ473706
		Hap_3	2	0.11111	PQ473701, PQ473708
		Hap_4	1	0.05555	PQ473693
		Hap_5	1	0.05555	PQ473694
		Hap_6	2	0.11111	PQ473695, PQ473698
		Hap_7	3	0.16666	PQ473696, PQ473707, PQ473710
	**ND4**				
	**321** **(n = 17)**	Hap_1	1	0.058824	PQ516178
		Hap_2	1	0.058824	PQ516172
		Hap_3	1	0.058824	PQ516177
		Hap_4	2	0.117647	PQ516179, PQ516186
		Hap_5	5	0.294118	PQ516180, PQ516181, PQ516182, PQ516184, PQ516187
		Hap_6	1	0.058824	PQ516183
		Hap_7	2	0.117647	PQ516185, PQ516188
		Hap_8	2	0.080547	PQ516173, PQ516175
		Hap_9	1	0.058824	PQ516176
		Hap_10	1	0.058824	PQ516174
	**Combined COI-ND4**				
	**980** **(n = 17)**	Hap_1	1	0.058824	(PQ473700 + PQ516178)
		Hap_2	2	0.080547	(PQ473703 + PQ516181), (PQ473706 + PQ516184)
		Hap_3	1	0.058824	(PQ473705 + PQ516183)
		Hap_4	3	0.17647	(PQ473704 + PQ516182), (PQ473702 + PQ516180),(PQ473709 + PQ516187)
		Hap_5	2	0.080547	(PQ473701 + PQ516179), (PQ473708 + PQ516186)
		Hap_6	1	0.058824	(PQ473699 + PQ516177)
		Hap_7	1	0.058824	(PQ473693 + PQ516172)
		Hap_8	1	0.058824	(PQ473695 + PQ516173)
		Hap_9	1	0.058824	(PQ473696 + PQ516174)
		Hap_10	1	0.058824	(PQ473697 + PQ516175)
		Hap_11	1	0.058824	(PQ473698 + PQ516176)
		Hap_12	2	0.080547	(PQ473707 + PQ516185), (PQ473710 + PQ516188)

**Table 3:** Positions of Parsimony Informative Sites (PIS) in the COI Gene of *Aedes aegypti* Sistan and Baluchistan populations. All polymorphic sites were parsimony informative, with no singleton mutations, using the most prevalent sequence as a reference.

Accession numbers (This study)	Positions											
5 7	1 9 2	2 5 8	2 6 1	2 7 0	3 2 7	4 8 3	4 9 2	5 0 4	5 2 5	6 0 3	6 5 4
PQ473700	C	A	C	T	A	A	A	A	A	C	A	A
PQ473703	.	.	.	.	.	.	.	.	.	T	.	.
PQ473705	.	.	.	.	.	.	.	.	.	.	.	.
PQ473706	.	.	.	.	.	.	.	.	.	T	.	.
PQ473704	.	.	.	.	.	.	.	.	.	.	.	.
PQ473702	.	.	.	.	.	.	.	.	.	.	.	.
PQ473701	.	.	.	.	.	.	.	.	.	T	G	.
PQ473699	.	.	.	.	.	.	.	.	.	.	.	.
PQ473693	.	G	.	.	G	C	G	G	.	.	.	G
PQ473694	.	.	.	.	.	C	.	.	.	T	.	.
PQ473695	T	G	T	C	G	C	G	G	G	.	.	G
PQ473696	T	G	T	C	G	.	G	G	G	.	.	G
PQ473697	.	.	.	.	.	.	.	.	.	T	.	.
PQ473698	T	G	T	C	G	C	G	G	G	.	.	G
PQ473707	T	G	T	C	G	.	G	G	G	.	.	G
PQ473708	.	.	.	.	.	.	.	.	.	T	G	.
PQ473709	.	.	.	.	.	.	.	.	.	.	.	.
PQ473710	T	G	T	C	G	.	G	G	G	.	.	G

**Fig 3:**
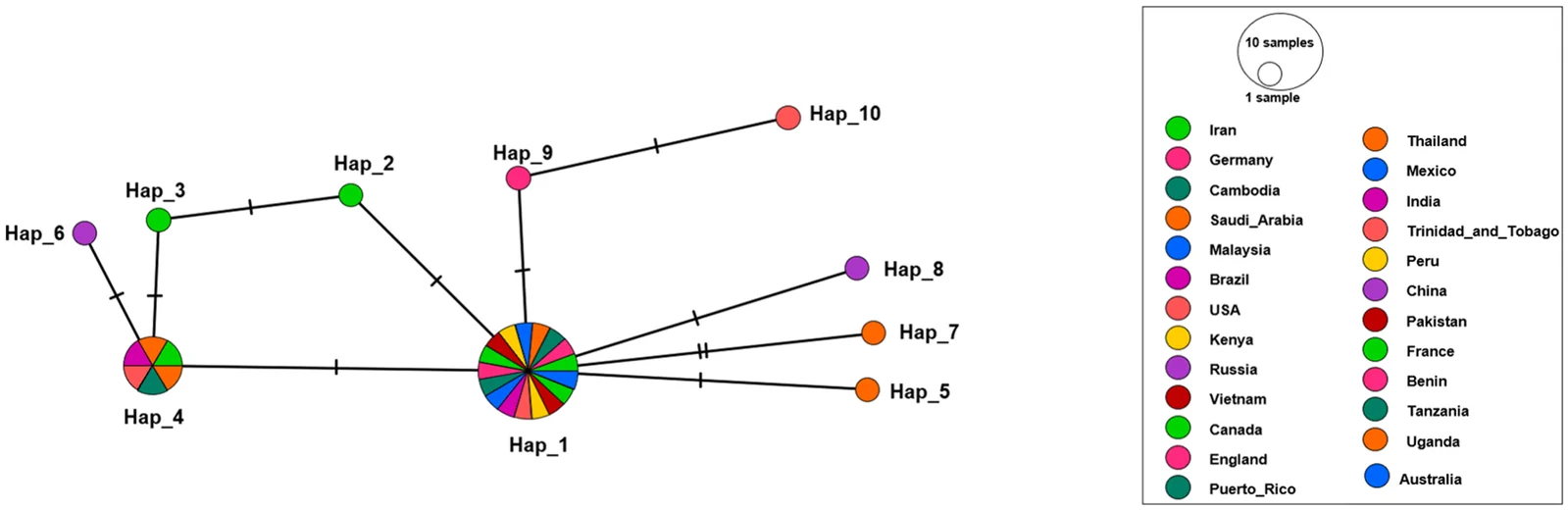
Haplotype network of *Aedes aegypti* based on COI sequences from 47 specimens (18 from Iran, 29 from global populations), showing 10 haplotypes. The network was constructed using the TCS method in PopART v.1.7. Each circle represents a distinct haplotype, with circle size proportional to haplotype frequency. Branch lengths are proportional to the number of mutational steps (1–2 steps between haplotypes), with each dash representing a single mutation. The largest circle (haplotype 1) comprises 28 sequences (59.6% of the total sample), including 10 Iranian specimens (PQ473699–PQ473706, PQ473708, and PQ473709), and is distributed across Oceania, Asia, Europe, North America, South America, and Africa. The second largest circle (haplotype 4) comprises 10 sequences (21.3% of the sample). The Benin haplotype (haplotype 9) is positioned between haplotype 10 (representing the ancestral *formosus* lineage) and the main haplotype 1, serving as a connecting link between the ancestral and derived lineages.

To further investigate the genetic relationships and phylogeographic structure of *Ae. aegypti* populations, a haplotype network was constructed using the COI sequences (597 bp) from the 18 Iranian specimens together with 29 COI sequences from global populations retrieved from GenBank (total n = 47). The analysis revealed 10 distinct haplotypes with a clear star-like network topology (Fig 3). Haplotype frequencies varied considerably, ranging from a single sequence to 28 sequences (59.6% of the total sample). Haplotype 1 formed the largest and most central node in the network, comprising 28 sequences (including 10 Iranian specimens, PQ473699 –PQ473706, PQ473708, and PQ473709) and were distributed across Oceania, Asia, Europe, North America, South America, and Africa. Haplotype 3 was the second largest (10 sequences, ~ 21% of samples), including four Iranian specimens (PQ473696, PQ473707, PQ473710, and PQ013114), and was positioned peripherally, separated from the haplotype 1 by a single mutational step.

Two Iran-specific haplotypes were identified: haplotype 2 represented by PQ473694 and PQ473693 (2 sequences) and haplotype 3 represented by PQ473695 and PQ473698 (2 sequences), forming medium-sized nodes in the network. The remaining six haplotypes were unique (each represented by a single sequence), corresponding to specimens from Russia, USA-F, Benin, Thailand, China, and Saudi Arabia. These formed the smallest circles at the network periphery. Notably, the Benin haplotype (haplotype 9) served as a connecting link between the haplotype 1 and haplotype 10 (representative of *formusos* lineage), suggesting that Africa may harbour an intermediate or ancestral haplotype.

### ND4 gene analysis

The complete alignment of all fragments spanned 321 nucleotides of the ND4 gene. Within this 321 bp region, ten haplotypes were identified (Table 2). Haplotype 5 was the most common (29.4%) and was detected in five specimens.

This gene exhibited 42 polymorphic sites (13.08%), including 14 parsimony-informative sites (33.3%) and 28 singleton variable sites (66.7%). Parsimony-informative sites were distributed at 14 positions, with one site (position 240) containing three variants and the remaining 13 positions (42, 45, 78, 93, 103, 123, 162, 171, 180, 204, 249, 291, and 297) containing two variants each (Table 4).

**Table 4:** Parsimony Informative Sites (PIS) in the ND4 Gene of *Ae. aegypti* Sistan and Baluchistan populations, compared to the Most Prevalent Sequence. 13 Sites with Two Variants (Highlighted in Blue) and One Site with Three Variants at Position 240 (Highlighted in Orange). Other variable or polymorphic positions are not parsimony-informative across two variants.

Accession numbers	Positions																																									
6	1 5	1 7	3 0	3 6	3 7	4 2	4 5	6 2	6 6	7 5	7 8	8 1	8 4	9 3	9 6	1 0 3	1 2 3	1 3 4	1 5 6	1 5 9	1 6 2	1 7 1	1 7 7	1 8 0	1 9 0	2 0 4	2 0 9	2 1 2	2 1 6	2 1 8	2 2 5	2 2 8	2 4 0	2 4 6	2 4 9	2 6 6	2 7 0	2 7 3	2 8 2	2 9 1	2 9 7
PQ516178	C	A	T	T	A	T	T	C	T	T	T	A	C	T	A	C	T	A	A	A	T	T	A	A	A	T	A	A	C	C	T	A	T	A	C	T	C	T	C	A	A	A
PQ516172	.	.	.	.	.	.	.	.	.	.	.	G	.	.	.	.	.	G	.	.	.	C	G	.	.	.	.	.	.	.	.	.	.	.	.	.	.	.	.	.	.	G
PQ516177	.	.	.	.	.	.	.	.	.	.	.	G	.	.	.	.	A	.	.	.	.	.	.	.	.	.	.	.	.	.	.	.	.	.	.	.	.	.	.	.	.	.
PQ516179	.	.	.	.	.	.	A	T	.	.	.	.	.	.	G	.	.	.	.	.	.	.	.	.	G	.	.	.	.	.	.	.	.	.	.	G	.	.	.	.	.	.
PQ516180	.	.	.	.	.	.	A	T	.	.	.	.	.	.	.	.	.	.	.	.	.	.	.	.	G	.	T	.	.	.	.	.	.	.	.	.	.	.	.	.	.	.
PQ516182	.	.	.	.	.	.	A	T	.	.	.	.	.	.	.	.	.	.	.	.	.	.	.	.	G	.	T	.	.	.	.	.	.	.	.	.	.	.	.	.	.	.
PQ516183	.	.	.	.	.	.	.	.	.	.	.	.	.	.	.	.	.	.	.	.	.	.	.	.	G	.	.	.	.	.	.	.	.	.	.	.	.	.	.	.	.	G
PQ516184	.	.	.	.	.	.	A	T	.	.	.	.	.	.	.	.	.	.	.	.	.	.	.	.	G	.	T	.	.	.	.	.	.	.	.	.	.	.	.	.	.	.
PQ516181	.	.	.	.	.	.	A	T	.	.	.	.	.	.	.	.	.	.	.	.	.	.	.	.	G	.	T	.	.	.	.	.	.	.	.	.	.	.	.	.	.	.
PQ516185	.	.	.	.	.	.	.	.	.	.	.	G	.	.	.	.	A	G	.	.	.	C	G	.	.	.	.	.	.	.	.	.	.	G	.	.	.	.	.	.	G	G
PQ516186	.	.	.	.	.	.	A	T	.	.	.	.	.	.	G	.	.	.	.	.	.	.	.	.	G	.	.	.	.	.	.	.	.	.	.	G	.	.	.	.	.	.
PQ516187	.	.	.	.	.	.	A	T	.	.	.	.	.	.	.	.	.	.	.	.	.	.	.	.	G	.	T	.	.	.	.	.	.	.	.	.	.	.	.	.	.	.
PQ516188	.	.	.	.	.	.	.	.	.	.	.	G	.	.	.	.	A	G	.	.	.	C	G	.	.	.	.	.	.	.	.	.	.	G	.	.	.	.	.	.	G	G
PQ516173	.	.	.	.	.	.	.	.	.	.	.	G	.	.	.	.	.	G	.	.	.	C	G	.	.	.	.	.	.	.	.	.	.	G	.	.	.	.	.	.	G	G
PQ516175	.	.	.	.	.	.	.	.	.	.	.	G	.	.	.	.	.	G	.	.	.	C	G	.	.	.	.	.	.	.	.	.	.	G	.	.	.	.	.	.	G	G
PQ516176	.	.	.	.	.	.	.	.	.	.	.	G	.	.	.	.	.	G	.	.	.	.	.	.	.	.	.	.	.	.	.	.	.	G	.	.	.	.	.	.	G	G
PQ516174	T	T	A	A	T	C	.	T	A	C	A	.	T	C	.	A	.	.	G	T	C	C	.	T	.	A	.	T	T	T	A	G	A	T	T	.	T	A	T	T	.	.

**Fig 4:**
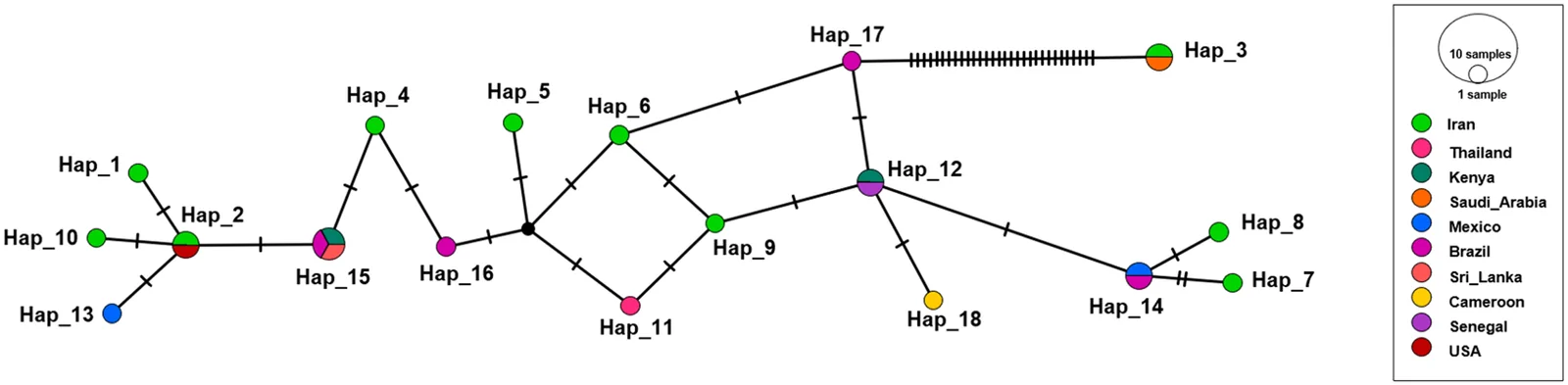
Haplotype network of *Aedes aegypti* based on ND4 sequences from 31 specimens (17 from Iran, 14 from global populations), showing 17 haplotypes. The network was constructed using the TCS method in PopART v.1.7. Each circle represents a distinct haplotype, with circle size proportional to haplotype frequency. Branch lengths are proportional to the number of mutational steps (1–2 steps between haplotypes), with each dash representing a single mutation. An unnamed circle represents unsampled or hypothetical intermediate haplotypes inferred by the TCS algorithm. The largest circle (haplotype 1) comprised five Iranian sequences (29.4% of Iranian samples). One of the most peripheral haplotypes (haplotype 3 from Iran and Saudi Arabia) was connected via a long branch (approximately 30 mutational steps) to the network, possibly representing the root.

A haplotype network was constructed for the ND4 gene (287 bp) using 31 sequences (17 Iranian specimens and 14 global sequences) with the TCS method in PopART. The network revealed 18 distinct haplotypes with complex reticulate relationships, including two loops (Fig 4). The largest circle (haplotype 1) comprised five Iranian sequences (29.4% of Iranian samples). One of the most peripheral haplotypes (haplotype 3 from Iran and Saudi Arabia) was connected via a long branch (approximately 30 mutational steps) to the network, possibly representing the root. This complex topology, including a central hub with long branches radiating to peripheral haplotypes, suggests a more ancient or complex demographic history for ND4 compared to COI.

### Combined COI–ND4 analysis

After concatenating the COI and ND4 sequences using DAMBE [39], we performed a combined mitochondrial sequence analysis (n = 17, total length 980 bp). In the combined COI–ND4 dataset, 12 haplotypes were identified (Table 2), encompassing 54 variable sites (5.5%), of which 26 (48.2%) were parsimony-informative and 28 (51.8%) were singleton variable sites.

### Variation in amino acid sequences

In the COI gene, all nucleotide substitutions were synonymous, resulting in identical amino acid sequences across all specimens. In contrast, the ND4 gene exhibited ten variable amino acid positions (9.4%) among the analysed sequences (S1 Fig).

Notably, the *Ae. aegypti* isolate from location 1 (GenBank accession PQ516174) was the most variable, showing nine non-synonymous substitutions among the 106 amino acid positions analyzed. The remaining specimens displayed only one variable site at position 73, where tyrosine (Y), an aromatic amino acid, was replaced by phenylalanine (F), increasing hydrophobicity. This substitution occurred in three sequences (accessions PQ516177, PQ516185, and PQ516188). The PQ516174 sequence showed the following non-synonymous substitutions at key positions: 19: Valine (V) → Isoleucine (I), 35: Methionine (M) → Leucine (L), 37: Valine (V) → Isoleucine (I), 38: Leucine (L) → Methionine (M), 44: Tyrosine (Y) → Phenylalanine (F), 63: Tryptophan (W) → Arginine (R), 87: Methionine (M) → Leucine (L), 95: Asparagine (N) → Serine (S), 102: Isoleucine (I) → Leucine (L). Except for these three specimens, the ND4 amino acid sequences were conserved, showing no additional non-synonymous substitutions.

### Phylogenetic analysis

#### Phylogenetic analysis of the COI gene.

For the phylogenetic analysis, 48 *Ae. aegypti* COI sequences were aligned, including 30 sequences from GenBank and 18 sequences from eight localities in the study area (S1 Table). The COI sequence of *Ae. albopictus* (GenBank IDs: NC006817) served as the outgroup.

**Fig 5:**
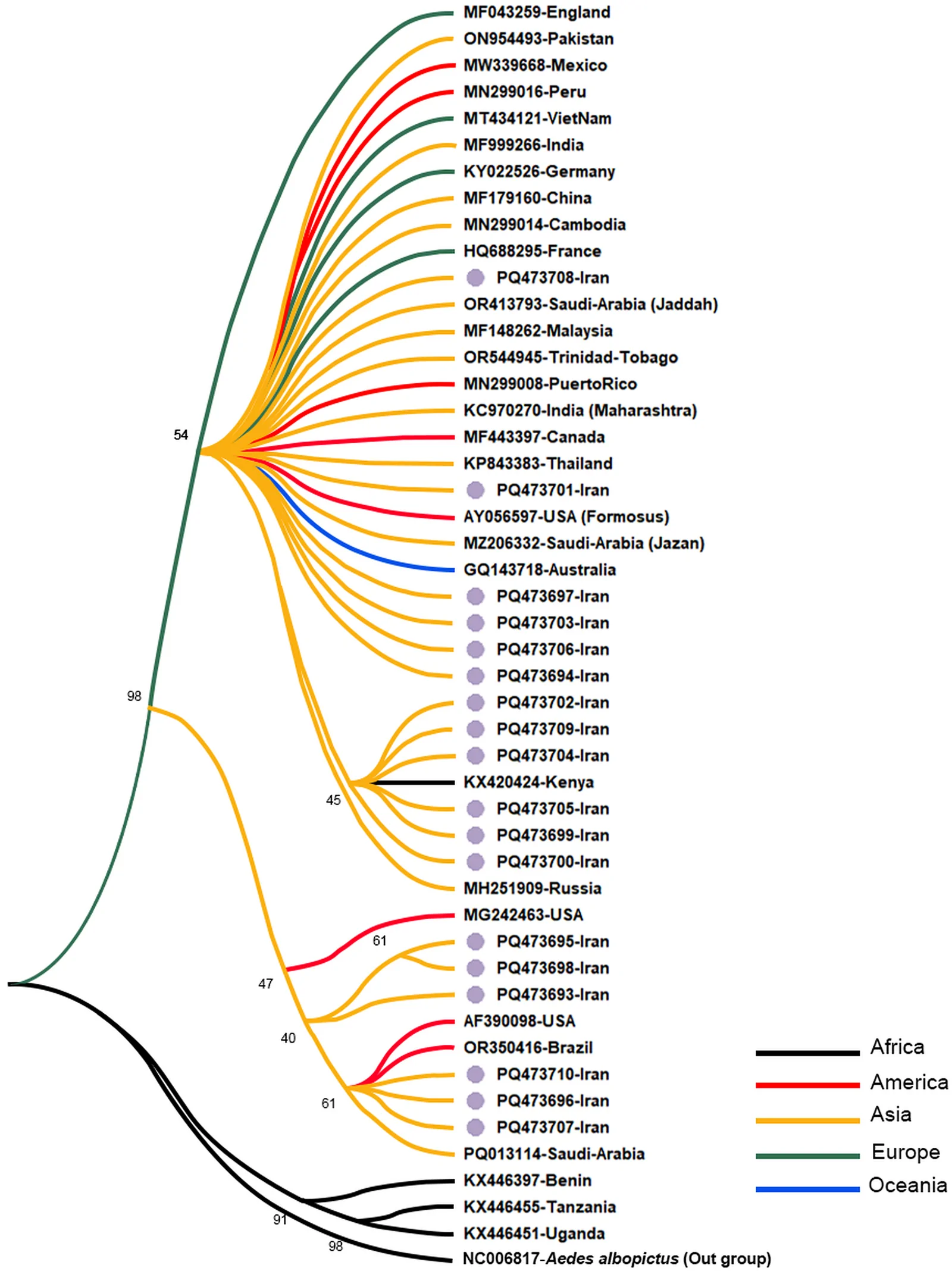
Bootstrapped Maximum Likelihood (ML) phylogenetic tree of *Aedes aegypti* based on the COI (659 bp) gene. The tree includes 18 sequences from this study (marked with pink circles) and 29 sequences from GenBank. Bootstrap values >30% are shown at the nodes. Each sequence is labeled with its GenBank ID and country of origin.

The Maximum Likelihood tree (Fig 5) revealed a basal clade with strong bootstrap support (95%), comprising sequences from West Africa (Benin) and East Africa (Tanzania and Uganda). Two derived clades diverged from this basal group, containing sequences from Iran, the Americas, Europe, Asia, and Australia. Iranian specimens were distributed across these derived clades, some grouped with Latin American, U.S., Asian, European, and Australian sequences, while others clustered with U.S., Brazilian, and Saudi Arabian isolates.

Most nodes showed strong support (bootstrap > 70%), though a few exhibited moderate values (40–50%), possibly reflecting limited sequence divergence or insufficient phylogenetic signal within this region of the COI gene. To enhance lineage resolution, future work could integrate additional mitochondrial or nuclear loci and broaden sampling across key geographic areas. Despite minor uncertainties, the phylogeographic structure was well-supported, and the inclusion of full-length COI sequences from GenBank provided a robust framework for intercontinental comparisons.

### Phylogenetic analysis of the ND4 gene

For the phylogenetic analysis, 31 *Ae. aegypti* ND4 sequences were aligned, including 14 sequences from GenBank and 17 sequences from eight localities within the study region (S1 Table). The ND4 sequence of *Ae. albopictus* (GenBank IDs: NC006817) served as the outgroup.

**Fig 6:**
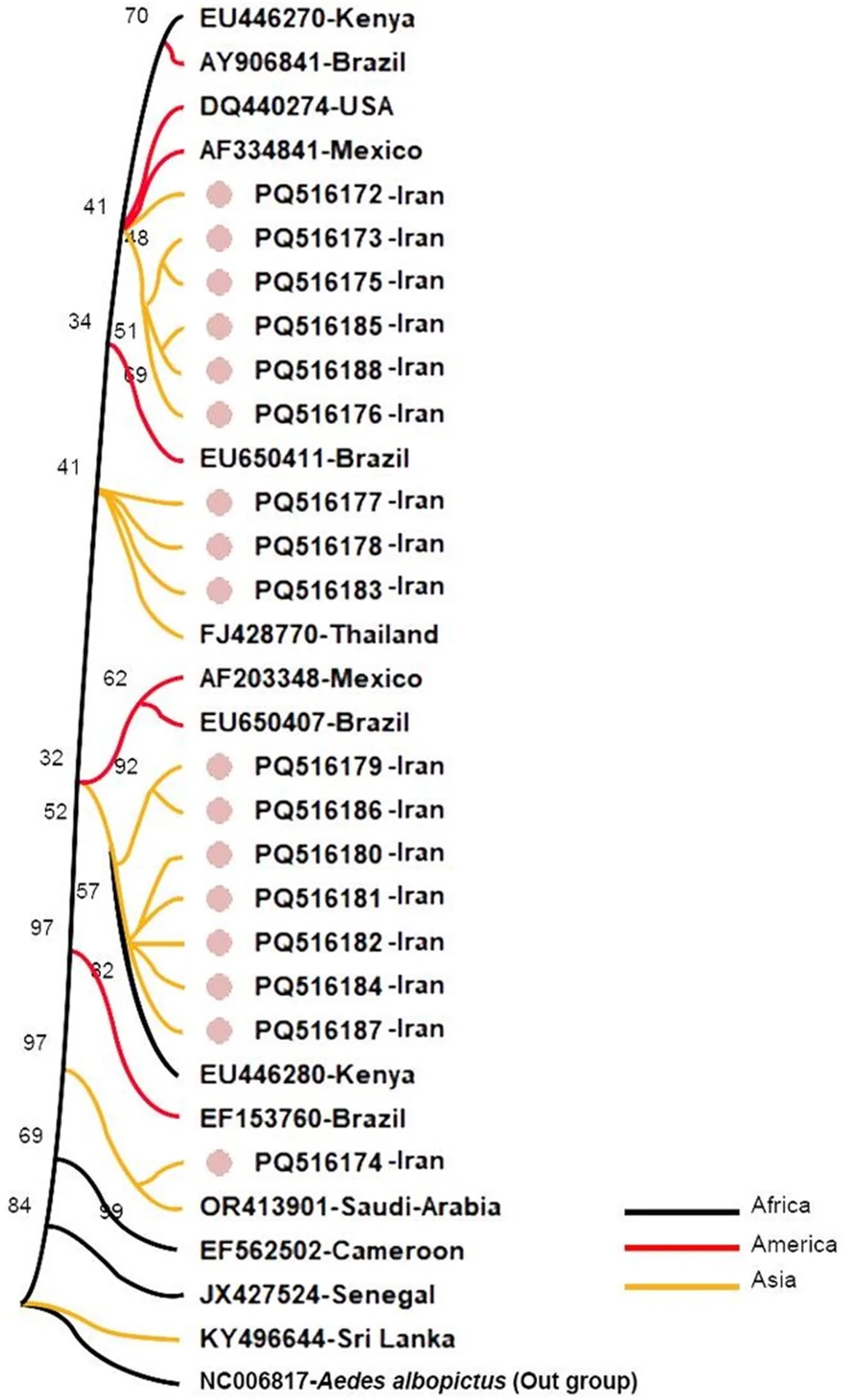
Bootstrapped Maximum Likelihood (ML) phylogenetic tree of *Aedes aegypti* based on the ND4 (321 bp) gene. The tree includes 17 sequences from this study (marked with pink circles) and 14 sequences from GenBank. Bootstrap values >30% are shown at the nodes. Each sequence is labeled with its GenBank ID and country of origin.

The Maximum Likelihood tree based on ND4 sequences (Fig 6) revealed a basal clade with moderate to strong bootstrap support (69–97%), comprising sequences from Senegal (West Africa), Cameroon (Central Africa), Sri Lanka, and Saudi Arabia. Iranian specimens were not monophyletic; instead, they were distributed across several derived clades. Most Iranian sequences clustered with lineages from Latin America (Brazil and Mexico) or Africa, forming multiple distinct groups. Notably, one Iranian specimen (accession no. PQ516174) showed a close relationship with a Saudi Arabian sequence (bootstrap: 98%) near the tree’s basal region, while other Iranian specimens formed separate subclades within the derived lineages.

The limited availability of ND4 sequences in GenBank constrained the phylogeographic resolution, resulting in moderate support values for several nodes; 58% of branches exhibited bootstrap values below 50%. Despite these limitations, the ND4 phylogeny supports multiple genetic affiliations of *Ae. aegypti* in southeastern Iran, reflecting potential introductions from both African and American lineages.

### Phylogenetic analysis of COI + ND4 genes

After concatenating sequences using DAMBE, we performed a combined phylogenetic analysis of COI and ND4 sequences to infer the geographic and evolutionary relationships between Iranian and non-Iranian *Ae. aegypti* specimens. A total of 47 sequences were aligned, comprising 30 sequences from GenBank and 17 sequences from eight localities within the study area (S2 Table). The GenBank sequences were derived either from complete mitochondrial genomes or from concatenated COI and ND4 fragments of the same specimen. The COI and ND4 sequences of *Ae. albopictus* (GenBank IDs: NC006817) were used as outgroups. The concatenated alignment covered 767 nucleotides in total (S2 Table).

**Fig 7:**
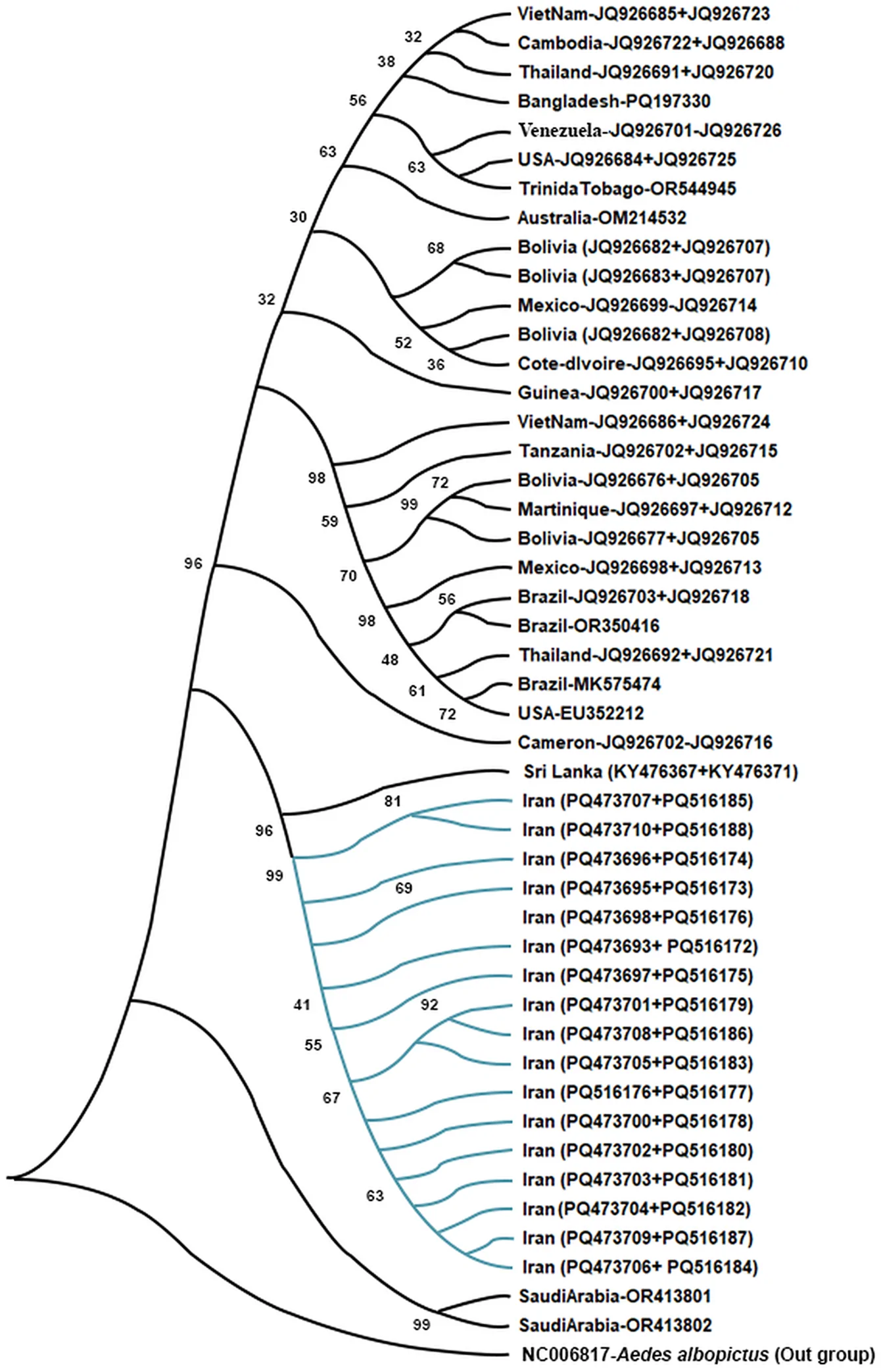
Bootstrapped Maximum Likelihood (ML) phylogenetic tree of *Aedes aegypti* based on the COI + ND4 (767 bp) gene combination. The tree includes 17 sequences from this study (marked with green squares) and 30 sequences from GenBank. Bootstrap values are shown at the nodes. Each sequence is labeled with its GenBank ID and country of origin.

The Maximum Likelihood tree based on the combined dataset (Fig 7) revealed a basal clade containing Saudi Arabian sequences, from which several derived clades emerged, representing specimens from Iran, North and South America, Australia, Africa, and Southeast Asia. Iranian specimens clustered with the Sri Lankan specimen and formed a monophyletic clade distinct from diverse global lineages from Asia, America, Australia, and Africa.

### Comparison of phylogenetic analyses (ND4, COI, and Combined ND4-COI)

The phylogenetic analyses based on the ND4, COI, and combined ND4–COI datasets consistently revealed two primary evolutionary lineages: a basal clade and a derived clade. The Iranian *Ae. aegypti* specimens were distributed across both lineages, with several clustering closely with global collections from Latin America, Africa, and Southeast Asia, while others formed distinct subclades unique to Iran. Notably, in the ND4 tree, one Iranian specimen was positioned near the basal clade, suggesting a possible ancestral lineage or an independent introduction event within the Iranian populations.

## Discussion

This study provides the first evidence of the presence and genetic structure of *Ae. aegypti* in southeastern Iran. The results demonstrate clear genetic structuring, revealing two distinct mitochondrial lineages of this species within the region. The COI-based phylogenetic tree (Fig 5) revealed two distinct mitochondrial lineages among global *Ae. aegypti* populations. The upper lineage comprised specimens from Iran, Asia, Europe, North America, South America, Australia, Russia, and Saudi Arabia. The lower lineage was exclusively composed of specimens from Africa (Benin, Tanzania, and Uganda). All Iranian specimens fell within the upper lineage, indicating closer genetic affinity with Asian, European, American, and Australian populations than with the African lower lineage.

To further elucidate the genealogical relationships among haplotypes, a haplotype network was constructed based on COI sequences from 47 specimens (18 Iranian and 29 global sequences) using the TCS method in PopART. The network revealed a star-like topology with a dominant central haplotype (haplotype 1) and rare peripheral haplotypes separated by 1–2 mutational steps. This pattern is consistent with a recent population expansion following a bottleneck or a recent common ancestor with subsequent radiation [40]. The widespread distribution of haplotype 1 across six continents indicates high haplotype sharing and suggests either natural long-distance dispersal, human-mediated transport (e.g., via maritime trade routes), or incomplete lineage sorting due to a recent common ancestor. Similar patterns have been reported for *Ae. aegypti* globally, reflecting the species’ association with human habitats and historical spread during colonial trade routes [10,11,41]. Haplotype 1, the largest and most centrally positioned in the network, comprised 28 sequences (59.6% of the total sample), including 10 Iranian specimens, and was distributed across Oceania, Asia, Europe, North America, South America, and Africa. Notably, the Benin haplotype (haplotype 9) was positioned between haplotype 1 and haplotype 10 (*Ae. aegypti formosus*), the sylvatic lineage primarily found in sub-Saharan Africa [40]. Haplotype 10 occupied a peripheral position in the network, suggesting its role as an ancestral or root lineage. The positioning of haplotype 9 as a bridge between haplotype 10 (*formosus*) and haplotype 1 (*aegypti*) lineage thus provides additional visual evidence for the African origin of global *Ae. aegypti* populations, with the *formosus* lineage giving rise to the derived domestic *aegypti* lineage that subsequently spread globally [11,40,41].

For the ND4 gene, a separate haplotype network was constructed using 31 sequences (17 Iranian and 14 global sequences). The network revealed 26 distinct haplotypes with a more complex topology compared to COI, including two nodes and two loops. Haplotype 3 included a Saudi Arabian specimen and an Iranian specimen (PQ516174) and occupied a peripheral position, separated by almost 30 mutations from the main network, suggesting it may represent a possible root. This complex topology, including multiple loops and a central hub with long branches radiating to peripheral haplotypes, suggests that ND4 captures deeper evolutionary history or more recent demographic events than COI.

The observed genetic diversity in this region may reflect the province’s strategic geographic and economic position, particularly Chabahar, which lies along a major maritime trade corridor connecting the Indian Ocean and Oman Sea to the Iranian mainland. Frequent ship traffic and cargo exchanges could have facilitated multiple introductions of *Ae. aegypti* into the area. The distribution of Iranian specimens across the dominant haplotype 1, haplotype 4, two Iran-specific haplotypes (in COI), and some peripheral nodes supports the hypothesis of multiple introduction events or contributions from distinct source populations.

Global population studies based on nuclear and mitochondrial markers, including microsatellites, single nucleotide polymorphisms (SNPs), whole-genome sequencing, and mtDNA genes, have identified two main genetic lineages of *Ae. aegypti*. Our analysis of ND4, COI, and concatenated datasets revealed two mitochondrial lineages within the study area, consistent with findings from Sri Lanka [21], Bolivia [42], Brazil [43], Venezuela [44], Colombia [45], Argentina [46], and Peru [36]. The haplotype network complements these findings by demonstrating that the two lineages are connected through the Benin haplotype (Africa), reinforcing the hypothesis that Africa may represent a source population or a bridge for the global spread of *Ae. aegypti* [40].

Although previous population genetic studies suggest that genetic structure can influence dengue transmission potential [12–15], the lack of detailed entomological surveillance and insecticide-use data at our sampling sites limits our ability to infer local differences in vector competence or resistance. Nevertheless, informal health reports indicate that dengue cases are considerably more frequent in Sistan and Baluchistan than in neighboring Hormozgan Province. This disparity may reflect climatic conditions, differences in healthcare and vector control infrastructure, socioeconomic factors, and the higher presence of migrants from Afghanistan and Pakistan who may serve as virus reservoirs. Furthermore, the coexistence of two distinct *Ae. aegypti* subclades in our study area, compared with a single genetically uniform subclade in Hormozgan [47], could also contribute to these contrasting epidemiological patterns. Together, these observations highlight the need for integrated studies linking genetic, environmental, and epidemiological data to clarify how lineage composition and local conditions influence dengue transmission in southern Iran.

Sequence comparison showed that one Iranian specimen (GenBank ID: PQ516174) clustered closely with Saudi Arabian specimens, suggesting possible introduction from neighboring countries. Given Chabahar’s coastal position and active maritime traffic through the Oman Sea, repeated introductions of *Ae. aegypti* are plausible. Comparative analysis of COI sequences from Hormozgan Province [47] showed that all ten sequences from that region were identical over a 606–642 bp fragment, matching haplotype 2 from our study over 611 bp. This result indicates potential gene flow or a shared origin between populations in the two provinces. The higher haplotype diversity observed in Sistan and Baluchistan may reflect multiple introductions, longer establishment, or greater genetic mixing, possibly contributing to the higher dengue incidence in this region. The haplotype network further supports this interpretation, as the 10 Iranian specimens sharing the major haplotype (haplotype 1) indicate genetic continuity with global populations, while the Iran-specific haplotypes suggest local diversification.

Because our study focused on vector detection and genetic profiling, we did not measure standard entomological indices such as the Positive House Index (PHI), Ovitrap Index (OI), or Ovitrap Density Index (ODI). Nonetheless, the absence of prior detections in the area and reports from neighboring regions strongly justified this investigation.

The phylogenetic analyses of COI, ND4, and the combined COI–ND4 datasets consistently placed Iranian *Ae. aegypti* populations within a single major mitochondrial lineage, although their internal clustering varied by marker. In the COI tree (Fig 5), all Iranian specimens fell within the upper lineage alongside samples from Asia, Europe, the Americas, Australia, and Saudi Arabia. The ND4 tree (Fig 6) showed greater resolution, with Iranian specimens distributed across several subclades; notably, one Iranian sample clustered near the root with a Saudi Arabian sequence. The combined COI–ND4 tree (Fig 7) provided the clearest phylogenetic signal, revealing a monophyletic clade comprising all Iranian samples together with a specimen from Sri Lanka, distinct from other global lineages. The haplotype network complements these phylogenetic trees by providing a more intuitive visualization of reticulate relationships, haplotype frequencies, and mutational steps among closely related haplotypes. While the phylogenetic trees resolve branching orders and evolutionary relationships, the network highlights the central role of the Australia haplotype and the low genetic divergence among global populations, reinforcing the conclusion that southeastern Iranian *Ae. aegypti* populations are part of a broader, recently expanded global lineage with ongoing gene flow.

Future genomic-scale studies, incorporating high-resolution molecular markers and integrated entomological monitoring, will be essential for tracking invasion routes, assessing adaptation, and developing targeted dengue prevention strategies in Sistan and Baluchistan Province and other high-risk regions of Iran.

## Conclusions

This study provides the first molecular evidence of *Ae. aegypti* in southeastern Iran, revealing high genetic diversity within the region. Haplotype network analyses of COI and ND4 genes further supported these findings: the COI network revealed a star-like topology with a dominant central haplotype (haplotype 1) shared among 10 Iranian specimens, indicating recent population expansion or high gene flow, while the ND4 network showed a more complex topology with Saudi-Arabia as a potential root, suggesting deeper evolutionary structure. Phylogenetic analyses consistently placed Iranian populations within a single major mitochondrial lineage, though ND4 sequences revealed subclade diversity and the combined COI–ND4 tree showed monophyly with a Sri Lankan specimen. These findings suggest multiple introductions or ongoing gene flow from diverse sources, which may influence vector competence and insecticide susceptibility. Given the province’s coastal trade connections and the high influx of migrants from neighboring dengue-endemic countries, the risk of dengue virus introduction and transmission is considerable. The results improve understanding of the species’ genetic structure in Iran and highlight the need for integrated genomic, ecological, and epidemiological studies to guide region-specific dengue surveillance and control strategies.

## Supporting information

S1 Table Mitochondrial COI sequences of global *Ae. aegypti* populations used for phylogenetic analysis. DS = direct submission [47–61].
(DOCX)

S2 Table Mitochondrial ND4 sequences of global *Ae. aegypti* populations used for phylogenetic analysis. DS = direct submission [21,40,41,62–68].
(DOCX)

S1 Fig Amino acid alignment of the mitochondrial NADH dehydrogenase subunit 4 (ND4) gene in *Aedes aegypti* Sistan and Baluchistan specimens, highlighting changes in the amino acid sequence. Dashes indicate identical amino acids to those in the top sequence.
(TIF)
