# Supplementary material for: Emergence and phylogeography of the dengue vector Aedes aegypti in Southeastern Iran
Source: PLoS Negl Trop Dis. 2026 Aug 20;20(8):e0014671. doi: 10.1371/journal.pntd.0014671 (PMC13577543; doi:10.1371/journal.pntd.0014671)
Supplement: Supplementary file 1 [file pntd.0014671.s001.docx]

| **Continent** | **Country (Isolate)** | **COI** | |
| --- | --- | --- | --- |
|  |  | **Accession numbers** | **Reference** |
| **Asia** | Iran | PQ473693 | This study |
|  |  | PQ473694 | This study |
|  |  | PQ473695 | This study |
|  |  | PQ473696 | This study |
|  |  | PQ473697 | This study |
|  |  | PQ473698 | This study |
|  |  | PQ473699 | This study |
|  |  | PQ473700 | This study |
|  |  | PQ473701 | This study |
|  |  | PQ473702 | This study |
|  |  | PQ473703 | This study |
|  |  | PQ473704 | This study |
|  |  | PQ473705 | This study |
|  |  | PQ473706 | This study |
|  |  | PQ473707 | This study |
|  |  | PQ473708 | This study |
|  |  | PQ473709 | This study |
|  |  | PQ473710 | This study |
|  | Saudi Arabia (Jeddah) | OR413793 | [47] |
|  | Saudi Arabia (Jazin) | MZ206332 | DS |
|  | Saudi Arabia | PQ013114 | DS |
|  | India (Maharashtra) | KC970270 | [48] |
|  | India | MF999266 | DS |
|  | Pakistan | ON954493 | DS |
|  | China | MF179160 | DS |
|  | Vietnam | MT434121 | DS |
|  | Malaysia | MF148262 | [49] |
|  | Cambodia | MN299014 | [50] |
|  | Russia | MH251909 | [51] |
|  | Thailand | KP843383 | [52] |
| **America** | Brazil | OR350416 | [53] |
|  | Trinidad- Tobago | OR544945 | DS |
|  | USA | MG242463 | [54] |
|  | USA | AF390098 | [55] |
|  | USA (Formosus) | AY056597 | [55] |
|  | Mexico | MW339668 | [56] |
|  | Peru | MN299016 | DS |
|  | Puerto Rico | MN299008 | DS |
|  | Canada | MF443397 | [57] |
| **Africa** | Uganda | KX446451 | [11] |
|  | Benin | KX446397 | [11] |
|  | Tanzania | KX446455 | [11] |
|  | Kenya | KX420424 | [11] |
| **Australia** | Australia | GQ143718 | [58] |
| **Europe** | England | MF043259 | [59] |
|  | Germany | KY022526 | [60] |
|  | France | HQ688295 | [61] |
