# Supplementary material for: Emergence and phylogeography of the dengue vector Aedes aegypti in Southeastern Iran
Source: PLoS Negl Trop Dis. 2026 Aug 20;20(8):e0014671. doi: 10.1371/journal.pntd.0014671 (PMC13577543; doi:10.1371/journal.pntd.0014671)
Supplement: Supplementary file 2 [file pntd.0014671.s002.docx]

| **Continent** | **Country (Isolate)** | **ND4** | |
| --- | --- | --- | --- |
|  |  | **Accession numbers** | **Reference** |
| **Asia** | Iran | PQ516172 | This study |
|  |  | PQ516173 | This study |
|  |  | PQ516174 | This study |
|  |  | PQ516175 | This study |
|  |  | PQ516176 | This study |
|  |  | PQ516177 | This study |
|  |  | PQ516178 | This study |
|  |  | PQ516179 | This study |
|  |  | PQ516180 | This study |
|  |  | PQ516181 | This study |
|  |  | PQ516182 | This study |
|  |  | PQ516183 | This study |
|  |  | PQ516184 | This study |
|  |  | PQ516185 | This study |
|  |  | PQ516186 | This study |
|  |  | PQ516187 | This study |
|  |  | PQ516188 | This study |
|  | Saudi Arabia | OR413901 | DS |
|  | Sri Lanka | KY496644 | [21] |
|  | Thailand | FJ428770 | [62] |
| **America** | Brazil | AY906841 | [63] |
|  |  | EU650411 | [40] |
|  |  | EF153760 | [64] |
|  |  | EU650407 | [40] |
|  | USA | DQ440274 | [65] |
|  | Mexico | AF334841 | [66] |
|  |  | AF203348 | [66] |
| **Africa** | Kenya | EU446280 | DS |
|  |  | EU446270 | DS |
|  | Cameroon | EF562502 | [67] |
|  | Senegal | JX427524 | [41] |
