# Supplementary material for: Emergence and phylogeography of the dengue vector Aedes aegypti in Southeastern Iran
Source: PLoS Negl Trop Dis. 2026 Aug 20;20(8):e0014671. doi: 10.1371/journal.pntd.0014671 (PMC13577543; doi:10.1371/journal.pntd.0014671)
Supplement: Supplementary file 3 [file pntd.0014671.s004.docx]

37. Please spell out NADH.

38. It would be better to use a clear and specific name for location.

43. Please replace ‘elsewhere; with a more specific and explicit expression,

59. Sistan and Baluchistan Province, Iran.

61. This mosquito species

94. please spell out Aaa and Aaf, and provide more ecological/evolutionary background on both groups.

97. You may want to specify what Ae. aegypti population is between Aaa and Aaf.

99. Please provide detailed (numeric) difference their competency between two subspecies.

102-108. Please provide examples of studies where these markers were used for Aedes mosquitoes. I suggest having Aedes aegypti examples.

110. Please provide county names.

111. How about establishments of *Aedes albopictus* in northern counties?

113-117. Not many people are familiar with the geography of regions other than their own. Please include a closer view around Iran to show neighboring countries with label in Figure 1. It would be better to include one additional step between the world map and the map of Iran. It will help readers to agree with your anticipation. Please contain all regional names you mentioned here in Figure 1.

119. Please provide the reasons why the genetic diversity and phylogeny of *Ae. aegypti* should be studied in this region.

130. You said the study site was across eleven counties, but in Figure 1, the ovitraps were located across more than 11 counties. It looks like more than 15 counties. Please check to make sure.

134. Please indicate the location of Lut Desert in Figure 1.

Table1. Each ovitraps were placed in different spots at each collection sites. So, technically, providing sixth decimal points is incorrect. Please check the width of the collection sites and provide coordinates with reliable decimal points.

162. Does ‘biweekly’ mean you collected samples two weeks after the setting?

165. Please explain more about “national and international vector surveillance guidelines” and cite them.

173. Please provide proper name of the stereomicroscope, and “(Olympus, Japan)” will be more accurate.

191. What is the volume of nuclease-free water?

Table S1 and S2. What does ‘DS’ stands for? Please spell it out or explain it in Table captions.

208. Why did you use *Culiseta* as an outgroup? As this study is to investigate genetic differentiations among populations of the same species, I think having other species belonging to the same genus is more reasonable. It might give inaccurate results if there are cryptic species within Iran *Aedes aegypti* populations. Please replace it with *Aedes albopictus*.

222. Please indicate Chabahar Konarak and Negur Counties in Figure 1.

Fig 2 and Fig 3. Please write full name of Stg. and make it italic.

265. How many samples did you use for genetic analysis?

Table 2. Please indicate what “*” means in the caption.

Reference: please use italic for species names

433-453. I don’t agree with your description of figure 7. Iranian samples only formed monophyly with Sri Lanka. Please revise this part. I partially agree with explanation with COI-based tree, not for ND4 and combined tree.

456-457. two distinct lineages in COI tree.

458-468. I cannot agree with this part at all. Diversity values should always be considered together with their standard deviations. For example here, the COI diversity of Iran is 0.00774, and the SD value is 0.00119. In that case, it may not be statistically different from the value in Brazil. Mean of ND4 diversity is 0.02948, but the SD is 0.00858, so it may not differ from other populations. You should either perform statistical analyses for this comparison or remove this statement.

470. Spell out SNPs please.

488. Please specify figure number.

510-517. I don’t agree with the interpretation. Please check my comments about 458-468.

Overall comments.

Thank you for providing your results as detailed as possible. However, I couldn’t agree with your conclusion. I recommend you conduct additional population genetics analyses, such as haplotype network. In addition, please provide more detailed information about methodology.
