# Supplementary material for: Emergence and phylogeography of the dengue vector Aedes aegypti in Southeastern Iran
Source: PLoS Negl Trop Dis. 2026 Aug 20;20(8):e0014671. doi: 10.1371/journal.pntd.0014671 (PMC13577543; doi:10.1371/journal.pntd.0014671)
Supplement: Supplementary file 4 [file pntd.0014671.s005.docx]

We thank the reviewers for their careful reading of our manuscript and for their constructive comments, which have helped us substantially improve the quality of our work. Below we address each comment point by point.

**Response to Reviewer #1**

**Comment** 37: Please spell out NADH.

**Response**: We thank the reviewer for this helpful suggestion. As requested, we have now spelled out the full name for NADH. On lines 37–38, the sentence has been revised to read: "Mitochondrial cytochrome oxidase I (COI) and nicotinamide adenine dinucleotide (NADH) dehydrogenase subunit 4 (ND4) genes…" Please also see lines 123–124.

**Comment** 38: It would be better to use a clear and specific name for the location.

**Response**: We thank the reviewer for this suggestion. The specific location name, Sistan and Baluchistan Province (SBP), is now provided on lines 36. The abbreviation "SBP" is used thereafter for brevity.

**Comment** 43: Please replace 'elsewhere' with a more specific and explicit expression.

**Response**: Thank you for this comment. As requested, on lines 44 we have replaced "elsewhere" with "Brazil, Mexico, Bolivia, and Venezuela" to provide a more specific and explicit expression. However, due to the comment of second reviewer we deleted genetic diversity analysis and this part of sentence was deleted as well.

**Comment** 59: Sistan and Baluchistan Province, Iran.

**Response**: Thank you. This has been corrected accordingly.

**Comment** 61: This mosquito species

**Response**: Thank you. "Species" has been added accordingly (line 65).

**Comment** 94: Please spell out Aaa and Aaf, and provide more ecological/evolutionary background on both groups.

**Response**: We thank the reviewer for this important suggestion. As requested, we have now spelled out the abbreviations Aaa (*Aedes aegypti aegypti*) and Aaf (*Aedes aegypti formosus*) on first use in the manuscript. In addition, we have expanded the ecological and evolutionary background for both subspecies in the revised manuscript (lines 99–111). Specifically, we have added information on their morphological differences, habitat preferences, host-seeking behavior, geographic distributions, and relative vector competence. We believe these additions substantially improve the clarity and completeness of the manuscript.

**Comment** 97: You may want to specify what *Ae. aegypti* population is between Aaa and Aaf.

**Response**: We thank the reviewer for this important clarification. Regarding intermediate or hybrid populations between Aaa and Aaf, we have now added references to the literature documenting such populations in East Africa. Gloria-Soria et al. (2016) confirmed using microsatellite markers that in urban East African settings, the two subspecies introgress freely, while remaining distinct in rural/forest settings (Please see line 109-110).

**Comment** 99: Please provide detailed (numeric) difference in their competency between the two subspecies.

**Response**: Thank you for your valuable comment. In response to your request to provide detailed (numeric) differences in competency between the two subspecies, we have now included specific quantitative data comparing Aaa and Aaf. These details have been added to the manuscript on lines 115–122.

**Comment** 102–108: Please provide examples of studies where these markers were used for *Aedes* mosquitoes. I suggest having Aedes aegypti examples.

**Response**: Thank you for your thoughtful comment. In accordance with your suggestion, we have now provided specific examples of studies where the mentioned markers were used for Aedes mosquitoes, with a particular focus on *Aedes aegypti*.

Specifically, we have added the following references (Refs. 16–18) to the manuscript on line 125, which demonstrate the application of these markers in *Aedes aegypti* populations:

16. de Lourdes Muñoz M, Mercado-Curiel RF, Diaz-Badillo A, Pérez Ramirez G, Black WC. Gene flow pattern among Aedes aegypti populations in Mexico. J Am Mosq Control Assoc. 2013;29:1–18. doi:10.2987/12-6267R.1

17. Fernando HSD, Hapugoda M, Perera R, Black Iv WC, De Silva BGDNK. Mitochondrial metabolic genes provide phylogeographic relationships of global collections of Aedes aegypti (Diptera: Culicidae). PLoS One. 2020;15:e0235430. doi:10.1371/journal.pone.0235430

18. Khater EIM, Baig F, Kamal HA, Powell JR, Saleh AA. Molecular Phylogenetics and Population Genetics of the Dengue Vector Aedes aegypti From the Arabian Peninsula. J Med Entomol. 2021;58:2161–2176. doi:10.1093/jme/tjab112

**Comment** 110: Please provide county names.

**Response**: Thank you for your comment. In response to your request, we have now provided the specific county names in the text accordingly (please see lines 132-133).

**Comment** 111: How about establishments of Aedes albopictus in northern counties?

**Response**: Thank you for this important question. In response, we have clarified that *Aedes albopictus* could not establish in either the southeastern counties nor the northern counties of Sistan and Baluchistan Province. However, this species has been found later in a northern province of Iran. The original sentence has been corrected accordingly to avoid any ambiguity. Please see the revised sentence on lines 134–139 of the updated manuscript.

**Comment** 113–117: Not many people are familiar with the geography of regions other than their own. Please include a closer view around Iran to show neighboring countries with labels in Figure 1. It would be better to include one additional step between the world map and the map of Iran. It will help readers agree with your anticipation. Please contain all regional names you mentioned here in Figure 1.

**Response:** Thank you for the suggestion. We agree that a closer view around Iran with labeled neighboring countries is helpful for readers unfamiliar with the region. Accordingly, we have removed the world map and now provide a clear regional map (Figure 1) showing Iran and all its neighboring countries, with all regional names mentioned in the text labeled directly on the map. While the reviewer suggested an intermediate step between the world map and the map of Iran, we believe the world map is not necessary for understanding our study, and the regional map alone serves the intended purpose more effectively by focusing readers' attention on the relevant geography.

**Comment** 119: Please provide the reasons why the genetic diversity and phylogeny of Ae. aegypti should be studied in this region.

**Response**: Thank you. We have now explicitly stated the reasons for studying the genetic diversity and phylogeny of *Ae. aegypti* in this region (see lines 145–149). Briefly, populations from different sources may vary in (1) vector competence for arboviruses and (2) insecticide resistance, both of which are critical for assessing outbreak risk and guiding control strategies.

**Comment** 130: You said the study site was across eleven counties, but in Figure 1, the ovitraps were located across more than 11 counties. It looks like more than 15 counties. Please check to make sure.

**Response:** Thank you. You are correct. Based on the new administrative division map, the study actually covered 18 counties, not 11. We have corrected the number in the text (see line 161) and ensured consistency with Figure 1.

**Comment** 134: Please indicate the location of Lut Desert in Figure 1.

**Response**: Thank you. The location of the Lut Desert has been added to Figure 1 and is now indicated by a brown spot, as described in the figure legend.

**Comment** (Table 1): Each ovitrap was placed in a different spot at each collection site. So, technically, providing six decimal points is incorrect. Please check the width of the collection sites and provide coordinates with reliable decimal points.

**Response**: Thank you for this careful observation. We agree that six decimal places would imply unrealistic precision given that ovitraps at each collection site were placed within a few meters to a maximum of 10 meters from the recorded point. However, the coordinates in Table 1 are actually presented to four decimal places (e.g., E 60.6440), which corresponds to a precision of approximately 11 meters at the equator. This is appropriate for the scale of our sampling design. Nevertheless, to avoid any misunderstanding, we have added the following note to the table legend: "Coordinates represent the approximate center of each collection site; ovitraps were placed within a 10-meter radius of this point."

**Comment** 162: Does 'biweekly' mean you collected samples two weeks after setting?

**Response**: Thank you for asking for clarification. By 'biweekly,' we mean once every two weeks. Specifically, samples were collected the day after ovitraps were set, and then once every 14 days thereafter. Please see line 185.

**Comment** 165: Please explain more about "national and international vector surveillance guidelines" and cite them.

**Response**: Thank you for this comment. We have revised the sentence to better explain the national vector surveillance guideline. Specifically, we have added a brief description of the guideline's recommendations, including the prioritization of high-risk areas such as border zones, customs checkpoints, seaports, and urban centers. The citation to the national guideline is provided as reference [32]. Please see lines 188–192 for the revised text.

**Comment** 173: Please provide the proper name of the stereomicroscope, and "(Olympus, Japan)" will be more accurate.

**Response**: Thank you. We have now provided the full proper name of the stereomicroscope, including the model number. The revised text reads: "Olympus SZX16 (Japan)". Please see line 197.

**Comment** 191: What is the volume of nuclease-free water?

**Response**: Thank you. The volume of nuclease-free water was 7.5 µL, as stated in line 215.

**Comment** (Tables S1 and S2): What does 'DS' stand for? Please spell it out or explain it in table captions.

**Response**: Thank you. 'DS' stands for "direct submission" and has been spelled out in the captions of Table S1 and Table S2 by adding "DS = direct submission."

**Comment** 208: Why did you use Culiseta as an outgroup? As this study is to investigate genetic differentiations among populations of the same species, I think having another species belonging to the same genus is more reasonable. It might give inaccurate results if there are cryptic species within Iran Aedes aegypti populations. Please replace it with Aedes albopictus.

**Response:** Thank you for this important methodological suggestion. We fully agree that outgroup choice is critical in phylogenetic analysis. We initially tested *Aedes albopictus* as a potential outgroup. However, in our preliminary analyses, the genetic proximity between *Ae. aegypti* and *Ae. albopictus* led to poor topological resolution, with *Ae. albopictus* sequences falling within the *Ae. aegypti* clade rather than serving as a clear outgroup.

Based on these results and a review of the literature, we have retained *Culiseta longiareolata* as the outgroup for the following reasons:

1. Close relationship between *Ae. aegypti* and *Ae. albopictus* reduces its suitability as an outgroup. *Culiseta longiareolata* belongs to a different genus within the Culicidae family, making it sufficiently distantly related to *Ae. aegypti* to serve as a proper outgroup. In contrast, *Ae. albopictus* is too closely related to *Ae. aegypti*, which can cause "long-branch attraction" artifacts or result in the outgroup being placed within the ingroup, as we observed in our preliminary analyses.

2. Precedent for using *Culiseta* or *Anopheles* as outgroups in *Ae. aegypti* studies. Some recent peer-reviewed studies on *Ae. aegypti* phylogenetics have successfully used more distantly related taxa as outgroups:

- Paksa et al. (2024, Parasites & Vectors), in the first report on the molecular phylogenetics of *Ae. aegypti* in Iran, used *Culiseta longiareolata* as the outgroup.

- Khan et al. (2025, Parasites & Vectors), in a population genetic study of *Ae. aegypti* in Pakistan, rooted the phylogenetic tree using outgroup taxa from related *Anopheles* species.

3. The genus *Aedes* is not monophyletic, making intra-genus outgroup choice complex. It is well-established in the literature that the genus *Aedes* is paraphyletic. Therefore, using a more distant outgroup like *Culiseta* provides a more stable and reliable root than a closely related but paraphyletically complex sister species.

For these reasons and based on our empirical data showing poor resolution when using *Ae. albopictus*, we are confident that *Culiseta longiareolata* is the appropriate outgroup for our analysis. We have clarified this justification in the Methods section (lines 243–248) to ensure transparency for readers.

**Comment** 222: Please indicate Chabahar, Konarak, and Negur Counties in Figure 1.

**Response**: Thank you. Chabahar and Konarak Counties are indicated in Figure 1 and are labeled as collection sites number 16 and 17. Negur is a part of Dashtyari County and is labeled as collection site 'a' in the figure.

**Comment** (Figures 2 and 3): Please write the full name of "Stg." and make it italic.

**Response:** The full name *Stegomyia* has been written in italics in Figures 2 and 3.

**Comment** 265: How many samples did you use for genetic analysis?

**Response**: Thank you for this comment. We have clarified the sample numbers in the text. Specifically, we now state that 18 specimens were used for COI sequencing, and ND4 sequences were obtained from 17 of those specimens (one amplification failure). Please see lines 267–270 for the revised text.

**Comment** (Table 2): Please indicate what "*" means in the caption.

**Response**: Thank you. The asterisk (*) indicates statistical significance at the 0.05 level. This has been added as a footnote to Table 2. However, based on the comment of reviewer number 2, this table was deleted,

**Comment** (References): Please use italics for species names.

**Response**: Thank you. We have checked all references and ensured that all species names are now italicized.

**Comment** 433–453: I don't agree with your description of Figure 7. Iranian samples only formed monophyly with Sri Lanka. Please revise this part. I partially agree with the explanation for the COI-based tree, not for ND4 and the combined tree.

**Response**: Thank you very much for your careful observation. We agree with you that in Figure 7 the Iranian samples only formed monophyly with Sri Lanka. Now we have corrected the text as follows: "The Maximum Likelihood tree based on the combined dataset (Fig. 7) revealed a basal clade containing Saudi Arabian sequences, from which several derived clades emerged, representing specimens from Iran, North and South America, Australia, Africa, and Southeast Asia. Iranian specimens clustered with the Sri Lankan specimen and formed a monophyletic clade distinct from diverse global lineages from Asia, Latin America, Australia, and Africa." Please see lines 410–412.

We have re-examined the ND4 tree (Fig. 6) and agree with you that the Iranian samples are not monophyletic. They are distributed across multiple derived clades, with most sequences grouping with Latin American or African lineages. One Iranian specimen (PQ516174) clusters with Saudi Arabia near the basal region. We have revised the text accordingly to accurately reflect these findings. Please see lines 387–392.

**Comment** 456–457: Two distinct lineages in COI tree.

**Response**: Thank you for the clarification. We have revised the text to explicitly describe the two distinct lineages observed in the COI tree (Fig. 5). The revised text now states that the upper lineage includes specimens from Iran, Asia, Europe, the Americas, Australia, Russia, and Saudi Arabia (Jadadah), while the lower lineage comprises specimens from Africa (Benin, Tanzania, and Uganda). All Iranian specimens belong to the upper lineage. Please see lines 369–372.

**Comment** 458–468: I cannot agree with this part at all. Diversity values should always be considered together with their standard deviations. For example, the COI diversity of Iran is 0.00774, and the SD value is 0.00119. In that case, it may not be statistically different from the value in Brazil. The mean ND4 diversity is 0.02948, but the SD is 0.00858, so it may not differ from other populations. You should either perform statistical analyses for this comparison or remove this statement.

**Response**: Thank you for this important statistical observation. We agree that comparisons of diversity values should account for standard deviations or be supported by statistical testing. We have therefore revised the text to remove any claims of "remarkably higher" diversity. Instead, we now simply report our observed values alongside those from other studies without making direct comparative claims, and we note that statistical testing would be required to confirm any differences. However, based on the comment of reviewer number 2, this data was deleted from the text,

**Comment** 470: Spell out SNPs, please.

**Response**: Thank you. SNPs has been spelled out as "single nucleotide polymorphisms (SNPs)." (Please see line 473).

**Comment** 488: Please specify the figure number.

**Response**: The figure number (Fig. 6) has been added. Please see line 515.

**Comment** 510–517: I don't agree with the interpretation. Please check my comments about 458–468.

**Response**: Thank you for your careful comments (458–468 and 510–517). We agree with your interpretation. Upon re-examination, we found that Iranian specimens are not distributed across both global lineages. We have revised the text to accurately reflect that all Iranian samples fall within a single major mitochondrial lineage across all three trees, while noting the internal subclade diversity observed in the ND4 tree and the monophyly of Iranian samples with Sri Lanka in the combined tree. Please see lines 511–525 for the revised text.

**Overall Comments**: Thank you for providing your results as detailed as possible. However, I couldn't agree with your conclusion. I recommend you conduct additional population genetics analyses, such as haplotype network. In addition, please provide more detailed information about methodology.

**Response:** We thank the reviewer for the constructive comment and for recommending the haplotype network analysis, which has substantially strengthened our manuscript. We have now conducted the requested analyses and revised the manuscript accordingly in abstract, M&M, results, and discussion sections. Below we detail the changes made.

1. Haplotype Network Analysis (COI)

We constructed a haplotype network for the COI gene (659 bp) using 47 sequences (18 Iranian specimens and 29 global sequences retrieved from GenBank) with the TCS method implemented in PopART v.1.7. The network (new Fig. S1) revealed 10 distinct haplotypes with a star-like topology. A dominant central haplotype (Australia) comprised 28 sequences (59.6% of the total sample), including 10 Iranian specimens, and was distributed across six continents. The Benin haplotype (Africa) served as a connecting link between the ancestral *Ae. aegypti* formosus lineage (USA-F) and the derived domestic aegypti lineage (Australia haplotype), supporting the African origin hypothesis. These findings have been integrated into the Results and Discussion sections. Please see lines 304-319 in result section and 431-452 in discussion section.

2. Haplotype Network Analysis (ND4)

We additionally constructed a haplotype network for the ND4 gene (321 bp) using 31 sequences (17 Iranian and 14 global sequences) with the TCS method in PopART v.1.7. The network (new Fig. S2) revealed 26 distinct haplotypes with a complex topology including three loops. The global nucleotide diversity (π) was 0.0328. The Saudi Arabian haplotype occupied the most peripheral position (root), with the Iranian specimen PQ516174 separated by a single mutational step. A central Brazilian sample (Brazil3) served as a major hub, connected to the root via a long branch (approximately 30 mutational steps). From Brazil3, three main branches emerged: one connecting to the root and PQ516174, and two connecting to networks linking samples from the Americas, Africa, Asia, and Iran. These findings have been integrated into the Results and Discussion sections. Please see lines 322-338 in result section and 453-464 in discussion section.

3. Methodology Details Added

We have expanded the Methods section to include detailed descriptions of the haplotype network analyses.

4. Revised Conclusion

Based on the haplotype network analyses and the previous comments of the respected reviewer, we have updated the Conclusion section as follows:

"This study provides the first molecular evidence of Ae. aegypti in southeastern Iran, revealing high genetic diversity within the region. Haplotype network analyses of COI and ND4 genes further supported these findings: the COI network revealed a star-like topology with a dominant central haplotype (Australia) shared among 10 Iranian specimens, indicating recent population expansion or high gene flow, while the ND4 network showed a more complex topology with Saudi Arabia as a potential root and Brazil as a central hub of diversity, suggesting deeper evolutionary structure. Phylogenetic analyses consistently placed Iranian populations within a single major mitochondrial lineage. These findings suggest multiple introductions or ongoing gene flow from diverse sources, which may influence vector competence and insecticide susceptibility."

We believe these revisions have substantially improved the manuscript and addressed the reviewer's concerns. We thank the reviewer again for the valuable suggestions.

---

**Response to Reviewer #2**

We thank Reviewer #2 for their positive assessment of the manuscript's interest, writing quality, and scholarship, as well as for their constructive criticism regarding the population genetics analyses. We have carefully considered all comments and have substantially revised the manuscript as detailed below.

**Comment** (Summary and General Comments): The authors report the first description of Aedes aegypti in Southeast Iran. This is important as it is a source of concern for the emergence of dengue in the country. The manuscript is interesting, well written, and worth publishing. However, this must be done only after a major revision. Indeed, part of the work, results, and conclusions are sound and can be kept. It is the morphological description and the phylogenetic analysis. The conclusion on the multiple introductions of populations through time is also very valid. However, the population genetics analysis and the allelic neutrality analysis with Fu and Li's F* test and Tajima's D test do not stand as they do not make sense. COI and ND4 are both mitochondrial genes. The mitochondrial DNA is a bacterial haploid chromosome with no alleles, no recombination, no exchange, and no introgression. Population genetics and neutrality tests are meant to be applied on diploid chromosomes to analyse population dynamics and allele distribution. They are not relevant for bacterial DNA exclusively transmitted passively through maternal inheritance. The differences noted by the authors in the genetic diversity of COI and ND4 only reflect their respective tolerance to drift. COI is under stronger purifying selection than ND4 and allows fewer variations. Mitochondrial genes are excellent phylogenetic and phylogeographic tools. The authors should reconsider their manuscript by removing the population genetics and allele neutrality tests and focusing on analysing deeper the meaning of the phylogenetic and phylogeographic data. They should then resubmit their manuscript.

**Response:** We thank the reviewer for this fundamental and highly valuable methodological critique. We fully agree with the reviewer's assessment. As requested, we have:

1. Removed all population genetics and neutrality test analyses (including Tajima's D, Fu and Li's F*, and related interpretations) from the manuscript, as these tests are indeed not appropriate for haploid, non-recombining mitochondrial DNA.

2. Retained and strengthened the phylogenetic and phylogeographic analyses, which are the appropriate uses of mitochondrial markers.

3. Added haplotype network analyses (as also requested by Reviewer #1) to provide a more appropriate population-level visualization of genealogical relationships.

4. Revised the title, abstract, M&M, Results and Discussion sections to focus exclusively on phylogenetic relationships, haplotype networks, and phylogeographic interpretations, without making claims about population genetics or selection.

5. Updated the Conclusion accordingly (see response to Reviewer #1 above).

We are grateful to the reviewer for this correction, which has substantially improved the scientific rigor of our manuscript.

---

**Response to Journal Requirements**

We have carefully addressed all journal requirements as follows:

1. CRediT author contributions: Completed for all authors, including Mohammad Ali Oshaghi, in the submission form.

2. Trademark symbols: Removed all instances of ® (page 9) and ™ (page 8) from the text.

3. Figure files: All main figures have been uploaded as separate .tif files.

4. Supplementary Tables: Removed from the manuscript file and uploaded as separate Supporting Information files with legends listed after the references.

5. Copyright and permissions:

- Figures 2 and 3 (photographs): We confirm that we are the photographers of these images.

- Figure 1 (map): The map was adapted from a previously published article in PLOS Neglected Tropical Diseases [citation]. As PLOS articles are published under the CC BY 4.0 license, adaptation is permitted provided the original source is credited. The figure legend now includes the appropriate attribution. No permission from a third party is required. The base layer was sourced from Natural Earth (public domain).

6. Financial Disclosure: Amended to state: "The funders had no role in study design, data collection and analysis, decision to publish, or preparation of the manuscript."

We believe all technical requirements have now been satisfied.

Sincerely,

The Authors
