# Supplementary material for: Emergence and phylogeography of the dengue vector Aedes aegypti in Southeastern Iran
Source: PLoS Negl Trop Dis. 2026 Aug 20;20(8):e0014671. doi: 10.1371/journal.pntd.0014671 (PMC13577543; doi:10.1371/journal.pntd.0014671)
Supplement: Supplementary file 5 [file pntd.0014671.s006.docx]

About Comment 208

I cannot agree with your response. The fact that *Ae. albopictus* fell within the *Ae. aegypti* ingroup is not justification for using *Culiseta* as an outgroup. Rather, it is strong evidence that something went wrong in your current analysis. *Aedes aegypti* and *Aedes albopictus* are distinct species, so under any properly conducted analysis, *Ae. albopictus* should fall outside *Aedes aegypti* group. There are two potential problems. Wrong samples or sequences were used, or unsound analysis methods were used.

Other studies using *Culiseta* as an outgroup does not validate your case. Those studies may have had entirely different reasons for selecting *Culiseta*, not having the same failure as your case. More importantly, there are published studies that have successfully used *Ae. albopictus* as an outgroup for *Ae. aegypti* phylogenetic analysis. (https://doi.org/10.1093/jme/tjaf112)

Additional Comment

The haplotype networks in Figure S1 and S2 do not follow the standard format for TCS-based network. Each node should be haplotype, not population, and each haplotype should have pie chart for geographic population. Please reconstruct the network.
