# Supplementary material for: Emergence and phylogeography of the dengue vector Aedes aegypti in Southeastern Iran
Source: PLoS Negl Trop Dis. 2026 Aug 20;20(8):e0014671. doi: 10.1371/journal.pntd.0014671 (PMC13577543; doi:10.1371/journal.pntd.0014671)
Supplement: Supplementary file 6 [file pntd.0014671.s007.docx]

**Response To Reviewer**

About Comment 208

I cannot agree with your response. The fact that *Ae. albopictus* fell within the *Ae. aegypti* ingroup is not justification for using *Culiseta* as an outgroup. Rather, it is strong evidence that something went wrong in your current analysis. *Aedes aegypti* and *Aedes albopictus* are distinct species, so under any properly conducted analysis, *Ae. albopictus* should fall outside *Aedes aegypti* group. There are two potential problems. Wrong samples or sequences were used, or unsound analysis methods were used.

Other studies using *Culiseta* as an outgroup does not validate your case. Those studies may have had entirely different reasons for selecting *Culiseta*, not having the same failure as your case. More importantly, there are published studies that have successfully used *Ae. albopictus* as an outgroup for *Ae. aegypti* phylogenetic analysis. (<https://doi.org/10.1093/jme/tjaf112>)

**Response to Reviewer**

We thank the reviewer for this critical observation. We agree that, *Ae. albopictus* should fall outside the *Ae. aegypti* clade, and its clustering within the ingroup in our initial analysis indicated a technical error.

We have thoroughly re-evaluated our data and identified the likely cause: a sample mislabeling or cross-contamination affecting the *Ae. albopictus* sequence. We have now replaced it with verified sequences from a morphologically identified *Ae. albopictus* specimen.

The revised phylogenetic analysis, performed with optimized methods, now correctly places *Ae. albopictus* as a distinct outgroup to Ae. aegypti, consistent with the reviewer's cited study and established taxonomy. The updated trees are presented in the revised manuscript.

We appreciate the reviewer's guidance in strengthening our analysis.

**Additional Comment**

The haplotype networks in Figure S1 and S2 do not follow the standard format for TCS-based network. Each node should be haplotype, not population, and each haplotype should have pie chart for geographic population. Please reconstruct the network.

**Response to Reviewer:**

We thank the reviewer for this important methodological comment. We agree that in TCS-based haplotype networks, each node should represent a distinct haplotype with pie charts illustrating the geographic composition of each haplotype.

In response, we have reconstructed both haplotype networks following the standard TCS format in PopART. Each circle now represents a single haplotype, with circle size proportional to haplotype frequency, and each circle is divided into pie sections representing the proportion of specimens from different geographic populations. The revised networks are now presented as Fig. 6 (COI) and Fig. 7 (ND4) in the main text (formerly Fig. S1 and S2). The corresponding figure legends have also been updated to reflect these changes.

We believe these revisions now meet the standard requirements for TCS network presentation.

We are grateful to the reviewer for their constructive feedback, which has significantly improved the quality of our manuscript.

Authors
